# AntiBMPNN: Structure‐Guided Graph Neural Networks for Precision Antibody Engineering

**DOI:** 10.1002/advs.202504278

**Published:** 2025-06-27

**Authors:** Ze‐Yu Sun, Jiayi Yuan, Divya Jaiswal, Jingxuan Ge, Tianjian Liang, Jiahui Wei, Jinghong Cao, Yulong Li, Xiaojie Chu, Yan Chen, Ying Xue, Wei Li, Tingjun Hou, Zhiwei Feng

**Affiliations:** ^1^ College of Pharmacology Sciences Zhejiang University of Technology Hangzhou 310014 P. R. China; ^2^ Department of Pharmaceutical Sciences School of Pharmacy University of Pittsburgh Pittsburgh PA 15261 USA; ^3^ College of Pharmaceutical Sciences Zhejiang University Hangzhou Zhejiang 310058 China; ^4^ College of Chemistry and Environmental Engineering Sichuan University of Science and Engineering Zigong 643000 P. R. China; ^5^ Department of Medicine Center for AIDS Research Division of Infectious Diseases School of Medicine University of Pittsburgh Pittsburgh PA 15261 USA; ^6^ Faculty of Pharmaceutical Sciences Shenzhen University of Advanced Technology Shenzhen 518107 P. R. China

**Keywords:** deep learning, message passing neuron networks (MPNNs), precision antibody engineering, sequence designs

## Abstract

Antibodies are crucial for medical applications, yet traditional methods for designing sequences are inefficient. This study introduces AntiBMPNN, an advanced deep‐learning framework that leverages an antibody‐specific 3D dataset, a fine‐tuned message‐passing neural network (MPNN), a frequency‐based scoring function, and AlphaFold 3 to achieve highly accurate antibody sequence design. AntiBMPNN surpasses ProteinMPNN with a perplexity of 1.5 and over 80% sequence recovery. Its scoring function, combined with AlphaFold 3, effectively prioritizes sequences based on structural recovery, positional stability, and biochemical or complex properties. Experimental validation highlights a 75% success rate in single‐point antibody design. AntiBMPNN consistently outperforms AbMPNN, AntiFold, and ProteinMPNN in designing complementarity determining regions (CDR) 1‐3, yielding stronger binding affinities. For CDR1 of huJ3 (anti‐HIV nanobody), it achieves a half maximal effective concentration (EC₅₀) of 9.2 nM (nanomolar), better than ProteinMPNN (135.2 nM) and AntiFold (59.3 nM), and comparable to AbMPNN (6.6 nM). For CDR2 of the D6 nanobody (targeting CD16), AntiBMPNN reaches 0.3 nM, outperforming AbMPNN (2.3 nM), AntiFold (0.7 nM), and ProteinMPNN (0.7 nM). In CDR3 of huJ3, it achieves 1.7 nM, surpassing AbMPNN (51.2 nM), with no detectable activity from AntiFold or ProteinMPNN. These findings confirm that AntiBMPNN‐designed sequences for J3 and D6 outperform the originals, highlighting its potential to improve therapeutic antibody design.

## Graphical abstract

AntiBMPNN, a deep learning framework that integrates antibody‐specific 3D data, MPNN, frequency‐based scoring, and AlphaFold 3, achieves superior antibody sequence design. In most cases, it outperforms ProteinMPNN, AntiFold, and AbMPNN in CDR design, demonstrating higher binding affinities and sequence recovery. These results highlight its potential for advancing therapeutic antibody development.

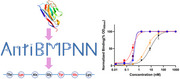

## 1 Introduction

In modern medicine, antibodies have become indispensable therapeutic agents, illustrating extraordinary efficacy across a broad spectrum of disease categories.^[^ ^1^ ^]^ This significance is proved by an exponentially expanding market forecasted to over $445 billion in the next half‐decade. Such promising expectation underscores the contribution of antibody‐derived therapies in the battle against a wide range of conditions‐encompassing malignancies, autoimmune disorders, and infectious diseases.

The conventional method of designing the sequence of antibodies, including full‐length antibodies and nanobodies, through an experimental process is both resource‐intensive and time‐consuming.^[^ ^2^, ^3^, ^4^ ^]^ This process typically involves immunizing animals, isolating specific B cells, and amplifying genes encoding antibody variable regions using polymerase chain reaction.^[^ ^5^ ^]^ Subsequent steps include cloning these genes into vectors to create antibody or nanobody libraries, followed by sophisticated screening techniques like phage display or yeast surface display to identify antigen‐specific candidates.^[^ ^6^ ^]^ The sequencing of selected clones further completes the arduous process, but additional steps are required, including extensive in vitro and in vivo assays to evaluate binding properties and functional activity. This is often followed by humanization and affinity maturation to enhance the therapeutic potential and ensure compatibility for clinical use. Despite the effectiveness of these experimental approaches, the challenges associated with their complexity, cost, and duration underscore the pressing need for alternative methods to expedite antibody sequence design and optimization.

Efforts in computational antibody design have focused on enhancing antibody functionality and specificity by strategically modifying specific residues within antibody structures, rather than altering the entire sequence. One approach involves grafting residues into existing antibody structures to optimize their binding properties.^[^ ^7^, ^8^, ^9^, ^10^, ^11^ ^]^ By introducing targeted amino acid substitutions in critical regions, such as the antigen‐binding sites, researchers can fine‐tune antibody characteristics to improve affinity and selectivity for target antigens without fundamentally changing the sequence. Another strategy involves sampling alternative native complementarity‐determining region (CDR) loops, essential for antigen recognition and binding.^[^ ^12^, ^13^, ^14^ ^]^ By exploring different conformations and sequences of CDR loops, computational methods identify optimal loop configurations that enhance antibody binding kinetics. This approach allows for the rational design of antibodies with improved antigen recognition capabilities by modifying residues on specific loops. Additionally, sequence design algorithms^[^ ^14^, ^15^, ^16^, ^17^ ^]^ like Rosetta^[^ ^18^, ^19^ ^]^ play a key role in refining antibody interactions with target epitopes through rational design principles. Rosetta utilizes computational modeling and optimization techniques to predict and optimize antibody–antigen interactions based on structural and energetic considerations. Rather than altering the antibody sequence, Rosetta facilitates the identification of key residues and interactions that can be optimized to enhance antibody binding and specificity for target epitopes. These computational methods enable the modification of specific residues within antibody structures to enhance functionality and specificity, offering a targeted and precise approach to antibody engineering without the need for wholesale sequence changes.

Recent breakthroughs in deep learning networks have introduced novel tools^[^ ^20^, ^21^, ^22^ ^]^ like Protein Message Passing Neural Networks (ProteinMPNN),^[^ ^22^ ^]^ which leverages deep learning techniques to predict and optimize protein structures with unprecedented precision. It's important to note that although ProteinMPNN offers promising capabilities, its sequence recovery—the ability to accurately predict and recover known antibody sequences from diverse datasets—is not ideal. The recovery rate is 52.4%^[^ ^22^ ^]^ for general proteins, but it decreases to ≈40% for antibody hypervariable regions.^[^ ^21^ ^]^ Despite these challenges, ProteinMPNN represents a significant advancement in computational protein design, offering a complementary approach to generate novel antibody candidates with tailored functionalities. AbMPNN,^[^ ^20^ ^]^ an antibody‐specific inverse folding model based on the ProteinMPNN architecture. Fine‐tuned on experimental and predicted antibody structures, AbMPNN sets new benchmarks for designability and amino acid sequence recovery, especially in the hypervariable CDRH3 loop. The model excels in conformational clustering and sequence recovery across CDR loops, demonstrating its potential to enhance antibody design and binding affinity. AntiFold^[^ ^21^ ^]^ excels in antibody design by leveraging an inverse folding approach tailored to antibody structures, resulting in superior sequence recovery and antibody–antigen binding affinity prediction compared to existing tools. It successfully integrates antigen information to refine predictions, particularly for complementarity‐determining regions, and synergizes with protein language models to guide antibody optimization. However, a notable drawback of both AbMPNN and AntiFold is the lack of extensive experimental validations, which limits their real‐world applicability and generalization beyond computational predictions. IgDesign,^[^ ^23^ ^]^ a deep learning method for antibody CDR design, marks the first antibody inverse folding model to be experimentally validated. IgDesign demonstrates its robustness by successfully designing binders for 8 therapeutic antigens, outperforming baseline models in both HCDR3 and HCDR123 designs. The model was tested through surface plasmon resonance screening and achieved high success rates, with some designs showing improved affinities compared to clinically validated reference antibodies. However, most of the designed sequences are lower than the baselines, which are directly obtained from the output of the algorithm. Additionally, IgDesign did not make its model or code publicly available, limiting further benchmarking and direct comparisons with other state‐of‐the‐art methods. The recent RFantibody pipeline introduces a novel strategy for antibody de novo design.^[^ ^24^ ^]^ It initially employs an antibody‐finetuned RFdiffusion model to generate backbone frameworks, subsequently utilizing ProteinMPNN to complete sequence predictions, and finally evaluates the newly designed antibody sequences using an antibody‐finetuned RoseTTAFold2 model. While this pipeline represents an innovative advancement, it remains challenged by high design complexity and significant validation costs.

Moving forward, efforts are underway to enhance the performance and robustness of these deep‐learning models in antibody engineering. This includes refining training datasets, optimizing model architecture, and integrating additional structural and sequence information to improve sequence recovery and overall predictive accuracy. To address the challenges outlined above, we propose AntiBMPNN, which leverages an antibody‐specific 3D training dataset, a fine‐tuned MPNN^[^ ^25^ ^]^ model, a novel frequency‐based scoring function, and AlphaFold 3 to design antibody sequences tailored for specific therapeutic targets. In‐silico evaluations demonstrate that AntiBMPNN outperforms ProteinMPNN in antibody sequence design, achieving higher accuracy (0.901), lower perplexity (1.511), and 60–80% sequence recovery. Experimental validation confirms a 75% success rate in stable single‐point designs. AntiBMPNN surpasses AbMPNN, AntiFold, and ProteinMPNN, achieving superior binding affinities in CDR design, including 9.2–99.6 nm for CDR1 and 1.7–418.9 nm for CDR3 of huJ3, a nanobody targeting HIV. AntiBMPNN‐designed CDR2 sequences for the CD16 nanobody (D6) achieve enhanced affinities, with the best at 0.3 nm. These findings confirm the efficiency and dependability of AntiBMPNN as a top‐tier solution for optimizing antibody sequences.

## 2 Results and Discussion

### 2.1 AntiBMPNN Framework and Training Performance

**Figure 1:**
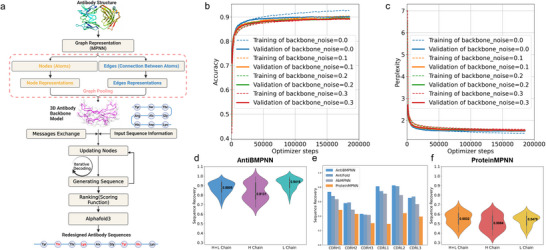
Overview of AntiBMPNN Framework and Training Performance. a) The framework of AntiBMPNN, which leverages graph neural network (GNN) to process and learn from complex antibody structures represented as graphs. By incorporating message‐passing layers, graph pooling operations, and specialized output layers, AntiBMPNN can effectively design and optimize antibody sequences. b) Accuracy of AntiBMPNN across different levels of backbone noise (0, 0.1, 0.2, and 0.3). The highest accuracy (0.927 for training and 0.901 for validation) is achieved at a noise level of 0, showing superior performance with minimal noise. c) Perplexity of AntiBMPNN at different backbone noise levels. The lowest perplexity (1.401 for training and 1.511 for validation) is observed when the backbone noise level is 0, indicating optimal model efficiency and prediction quality under these conditions. It should be noted that the figure only presents noise levels up to 0.3 and does not include higher levels of noise (0.4–1) for both (b) and (c). d) The average sequence recovery for the H+L chain (blue), H chain (purple), and L chain (green) in AntiBMPNN are 0.8809, 0.8131, and 0.9416, respectively. e) Sequence recovery across six CDR loops for different models shows that AntiBMPNN achieves the highest recovery in most regions, outperforming other methods. f) The average sequence recovery for the H+L chain (orange), H chain (red), and L chain (yellow) in ProteinMPNN are 0.5532, 0.5084, and 0.5479, respectively. The data for Figure 1d–f were derived from a testing dataset including 40 light chains, 40 heavy chains, and 40 paired antibody structures. For testing, the model weights that had the least backbone noise during training (0.0 for AntiBMPNN and 0.02 for ProteinMPNN) were chosen, using a sampling temperature (T) of 0.1. Specifically, the results shown in Figure 1b,c were generated from the training dataset, while Figure 1d–f were generated using the test dataset.

AntiBMPNN (antibody message passing neural network, **Figure** **1**a) processed antibody structures as graphs, encoding them with nodes representing atoms and feature vectors, and edges representing atomic interactions. Message‐passing layers propagated information between nodes, capturing hierarchical and spatial features. Graph pooling aggregated these features into a global summary. Trained on antibody 3D structure datasets, AntiBMPNN minimized a categorical cross‐entropy loss to reduce the difference between the predicted and actual amino acid sequences. Incorporating randomized decoding orders and attention mechanisms during training enhanced the model's ability to capture dependencies and contextual cues within antibody structures. The AntiBMPNN scoring function ranked candidate sequences based on frequency changes, incorporating sequence generation, comparison, and metadata like protein descriptors. Additionally. AlphaFold 3 was used to model the structures of the antigen complexed with the designed sequences ranked by the scoring function, selecting the model with the highest‐ranking score for experimental validation of the top designs.

Before evaluating AntiBMPNN's training performance with various backbone noise levels, we tested different parameter combinations using the auto‐adjust method. The parameters included “batch_size” (500–5000), “reload_data_every_n_epochs” (1–5), “dropout” (0.0–1), and “gradient_norm” (−1 to 4). Optimal performance was achieved with a batch_size of 1000, reload_data_every_n_epoch of 4, dropout of 0.2, and gradient_norm of −1.

Evaluation of training performance on a dataset of 6578 structures across different backbone noise levels revealed that AntiBMPNN achieved the highest accuracy (training: 0.927, validation: 0.901, Figure 1b) and lowest perplexity (training: 1.401, validation: 1.511, Figure 1c) at a noise level of 0. While Figure 1b,c only shows results for noise levels up to 0.3, we observed that as the noise increased further (0.4 to 1), both accuracies continued to decline, highlighting the model's sensitivity to noise. These results underscore the importance of data preprocessing and quality control to optimize AntiBMPNN's performance, particularly in antibody structure prediction for therapeutic development and biomedical research.

### 2.2 Sequence Recovery on Native Backbones Using AntiBMPNN

In further evaluating AntiBMPNN, we subjected it to a comprehensive test using a dataset comprising H+L‐chain, H‐chain, and L‐chain variants, totaling 120 distinct 3D structures. The evaluation revealed noteworthy median sequence recovery for AntiBMPNN across all residues: 88.09% for H+L‐chain structures, 81.31% for H‐chain structures, and 94.16% for L‐chain structures, as illustrated in Figure 1d. Conversely, when we assessed ProteinMPNN performance as shown in Figure 1f, the average sequence recovery was notably lower, standing at 55.32% for H+L‐chain, 50.84% for H‐chain, and 54.79% for L‐chain structures. A significant observation across all three cases of AntiBMPNN evaluation was the close correlation between sequence recovery and residue burial. This correlation was pronounced, with recovery ranging from 93% to 97% in the deep core to 85% to 91% on the surface of the structures.

In Figure 1e, we presented a comprehensive comparison of sequence recovery across six distinct CDR loops‐CDRH1, CDRH2, CDRH3, CDRL1, CDRL2, and CDRL3‐among four models: AntiBMPNN, AntiFold, AbMPNN, and ProteinMPNN. ProteinMPNN showed the lowest sequence recovery rates, with values of 0.4862, 0.4308, 0.3030, 0.2921, 0.4415, and 0.3938, respectively. AbMPNN improved upon this, achieving 0.6347, 0.4902, 0.4167, 0.7111, 0.6911, and 0.5660. AntiFold further improved performance in some loops, with values of 0.6786, 0.5893, 0.4205, 0.7465, 0.8142, and 0.6709, and it achieved the highest recovery for CDRH2 and CDRL3. AntiBMPNN, while not consistently best across all regions, achieved the highest recovery in four out of six loops‐CDRH1, CDRH3, CDRL1, and CDRL2‐with values of 0.7358, 0.5800, 0.4314, 0.8129, 0.8259, and 0.6526, respectively.

These results indicate that AntiBMPNN offers generally superior performance in sequence recovery, especially for the heavy‐chain CDRH1 and the light‐chain CDRL1 and CDRL2. Notably, CDRH3 remains the most difficult region across all models, with the lowest recovery rates (ranging from 0.3030 in ProteinMPNN to 0.4314 in AntiBMPNN), underscoring its design complexity. Additionally, all models tend to achieve higher recovery rates on the light‐chain loops than on the heavy‐chain loops, suggesting greater sequence conservation in the light chain.

### 2.3 Impact of Parameters on AntiBMPNN Performance

We also investigated the impact of training noise level and sampling temperature on sequence recovery. As illustrated in Figure S1a–d (Supporting Information), both AntiBMPNN and ProteinMPNN exhibited similar response trends to changes in these parameters. We observed a consistent decline in sequence recovery as training noise level and sampling temperature increased. For AntiBMPNN, the average lowest sequence recovery across the three test sets was ≈60%. In contrast, ProteinMPNN showed significantly lower recovery rates, with the average lowest reaching ≈30%. These comparisons suggest that higher levels of noise during training and elevated temperatures diminish the model's ability to accurately recover sequences. Conversely, increasing either or both parameters may be beneficial for generating new or diverse sequences.

Figure S2a,b (Supporting Information) illustrates the impact of sequence similarity between the test data and training data on sequence recovery. We assessed sequence similarity using a dataset of 40 H‐chain and 40 L‐chain crystal/cryo‐EM structures, plotting sequence recovery against sequence similarity. To quantify this relationship, we calculated the Pearson correlation coefficient (R) and added trendlines using linear regression. Our results revealed a clear correlation between increased sequence similarity and enhanced sequence recovery. This indicates that AntiBMPNN performs more reliably on sequences or motifs it has seen during training, which may reduce its generalizability when applied to novel or highly divergent sequences.

Furthermore, our findings suggest that maintaining consistency in either the backbone noise level or temperature parameter, particularly at a value of 0.5, is crucial for optimizing the performance of the AntiBMPNN model. Specifically, we conducted experiments in which we held one parameter‐either the backbone noise level or the sampling temperature‐constant at 0.5 while varying the other. This allowed us to isolate the individual impact of each parameter on sequence recovery. We found that maintaining one of these parameters at 0.5 consistently led to improved performance for both heavy‐chain (H‐chain) and light‐chain (L‐chain) antibodies. These results suggest that moderate, stable values for backbone noise or sampling temperature‐particularly ≈0.5‐are important for optimizing AntiBMPNN's sequence recovery performance.

Interestingly, a comparison of Figure S2a,b (Supporting Information) revealed that the correlations for the H‐chain (≈0.80) were higher than those for the L‐chain (≈0.65), despite the L‐chain demonstrating higher sequence recovery rates compared to the H‐chain. This suggests that while the L‐chain generally recovers sequences more effectively, the H‐chain has a stronger correlation between sequence similarity and recovery, highlighting differences in how sequence similarity affects recovery for different antibody chains.

### 2.4 Frequency‐Based Scoring Function and AlphaFold 3 for Sequence Selection

**Figure 2:**
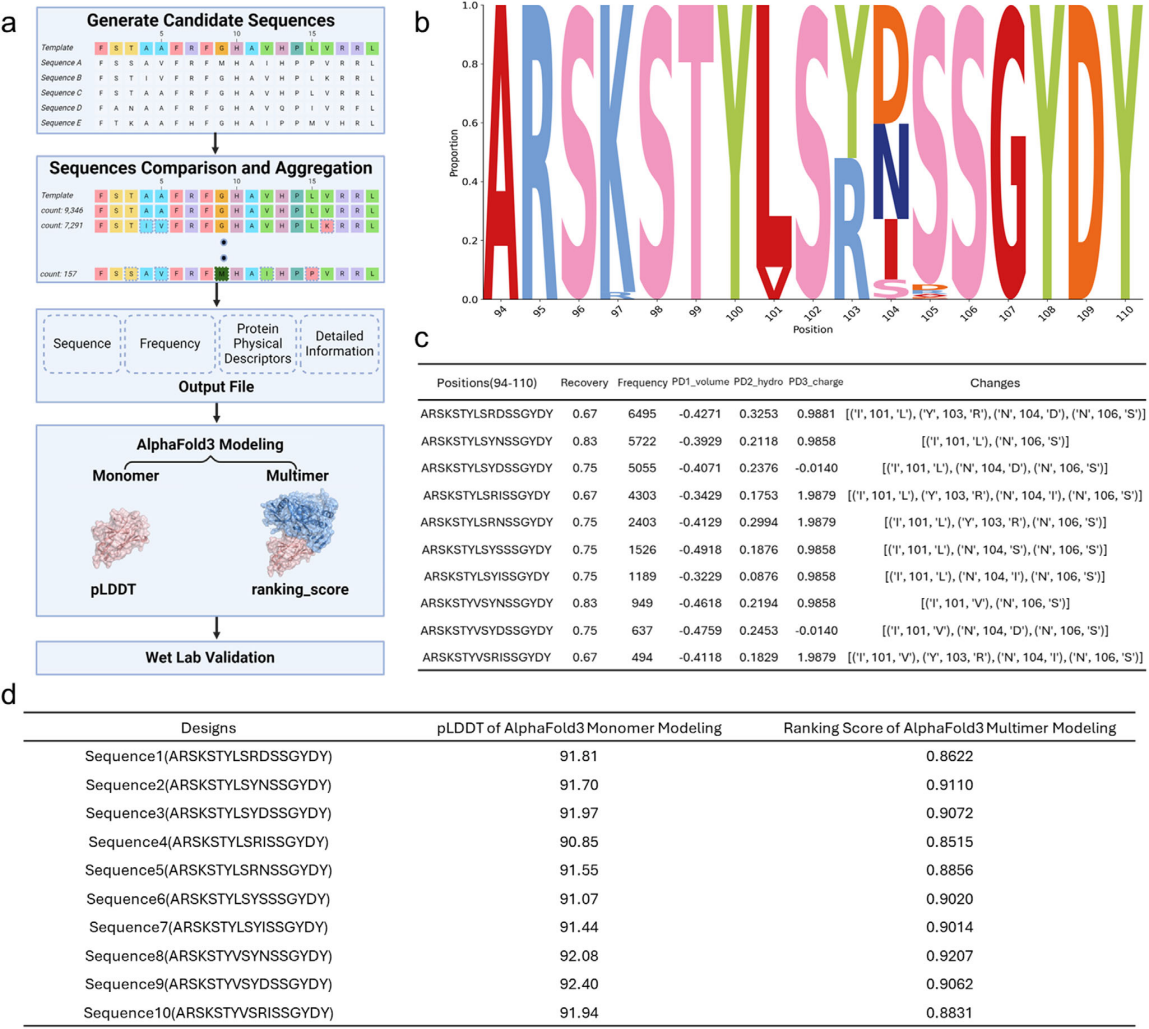
The integration of the scoring function in AntiBMPNN and AlphaFold 3 to rank candidates of each designed sequence. a) The flowchart of the frequency‐based scoring function prioritizes candidates through several key components, including the generation of candidate sequences, sequence comparison and aggregation, and other calculated metadata will be saved to the output file including frequency, protein physical descriptors, and other detailed information. b) A sequence logo example demonstrates how AntiBMPNN designs six positions—K97, I101, Y103, N104, S105, and N106—and the resulting frequency changes for each residue. The top‐ranked sequence, “ARSKSTYLSRDSSGYDY” (from positions 94 to 110), appeared 6495 times among the 32768 generated sequences. Notably, four positions (I101L, Y103R, N104D, and N106S) deviated from the original sequence of huJ3, resulting in a sequence recovery rate of 0.667. c) Another example illustrates the top 10 clusters of designed sequences as determined by the scoring function. In the middle of the three columns, “PD1_volume” represents the physical descriptors of residue volume, “PD2_hydro” represents the physical descriptors of hydrophilicity, and “PD3_charge” represents the charge properties at pH 7.4. d) AlphaFold 3 modeling metrics for designed sequences, including the best average pLDDT for monomer modeling and the best ranking score for antibody–antigen multimer modeling of each antibody sequence. The method section has detailed information on pLDDT and ranking scores.

AntiBMPNN designed the sequences with or without fixing any residue. For the output, AntiBMPNN initially generated many sequences, each of which might have multiple copies with the same sequence recovery but diverse scores, making it difficult for users to select candidates for further analysis. To overcome this limitation, a frequency‐based scoring function was designed to prioritize the candidates, which the flowchart of this algorithm was illustrated in **Figure** **2**a.

Figure 2b,c shows one example of how the scoring function worked. In the sequence design of huJ3, AntiBMPNN set six positions (K97, I101, Y103, N104, S105, and N106) in CDR3 to design and fixed all other positions. Among the generated sequences, totaling 32768, the scoring function grouped them into 147 clusters based on their sequences. Figure 2b shows that the top‐ranked sequence was “ARSKSTYLSRDSSGYDY” (from position 94 to 110 in CDR3), with 6495 occurrences. Compared with the original sequence of huJ3, four positions were changed, including positions I101L, Y103R, N104D, and N106S, with a sequence recovery of 0.67. Figure 2b,c depicted the frequency distribution of residue changes at the designated positions, providing insights into AntiBMPNN's sequence design strategy. Additionally, different chemical properties for each individual sequence were presented, facilitating the selection of candidates for further investigation.

To further evaluate the newly designed sequences ranked by the scoring function, AlphaFold 3 was used to generate both the antibody structure and its complex with the antigen. Each sequence was first modeled in monomer mode, generating five candidate models. The model with the highest pLDDT score was selected as the representative result of this sequence, indicating the likelihood of proper antibody self‐folding. Next, the antibody sequence was combined with its antigen sequence, such as gp120, to model the antibody–antigen complex. Five models were generated, and the model with the highest “ranking_score,” a newly introduced metric in AlphaFold3, was selected as the representative result, as shown in Figure 2d. The top one or two sequences with the highest pLDDT or ranking_score from each modeling method were considered for experimental validation. Detailed models’ information can be found in Table S1 (Supporting Information).

### 2.5 Single‐Point Sequence Design Using AntiBMPNN with Experimental Validation

We recently humanized the llama nanobody J3 into huJ3 to reduce its immunogenicity. This was achieved by grafting its CDRs (CDR1‐3) onto the closest human Ig germline variable domain (VH) scaffold, the VH3‐23 family. The resulting huJ3 differs by 13 amino acids from the original J3 nanobody. Notably, the CDR3 of huJ3 contains the motif “ARSKSTYISYNSNGYDY,” spanning residues 94 to 110. Our previous study^[^ ^26^ ^]^ identified the “STY” motif (residues 98 to 100) as crucial and highly sensitive to substitutions. Mutations at this site carry a high risk of compromising the antibody's binding ability. To design huJ3 sequences using AntiBMPNN, we first constructed the 3D structure of huJ3 based on the llama VHH nanobody J3 (nanoJ3, PDB: 7ri1), which shares 93% sequence identity. Additionally, we modeled the 3D structure of the huJ3‐gp120 complex, leveraging the crystal structure of nanoJ3 and gp120. This enabled a more precise exploration of sequence design.

**Figure 3:**
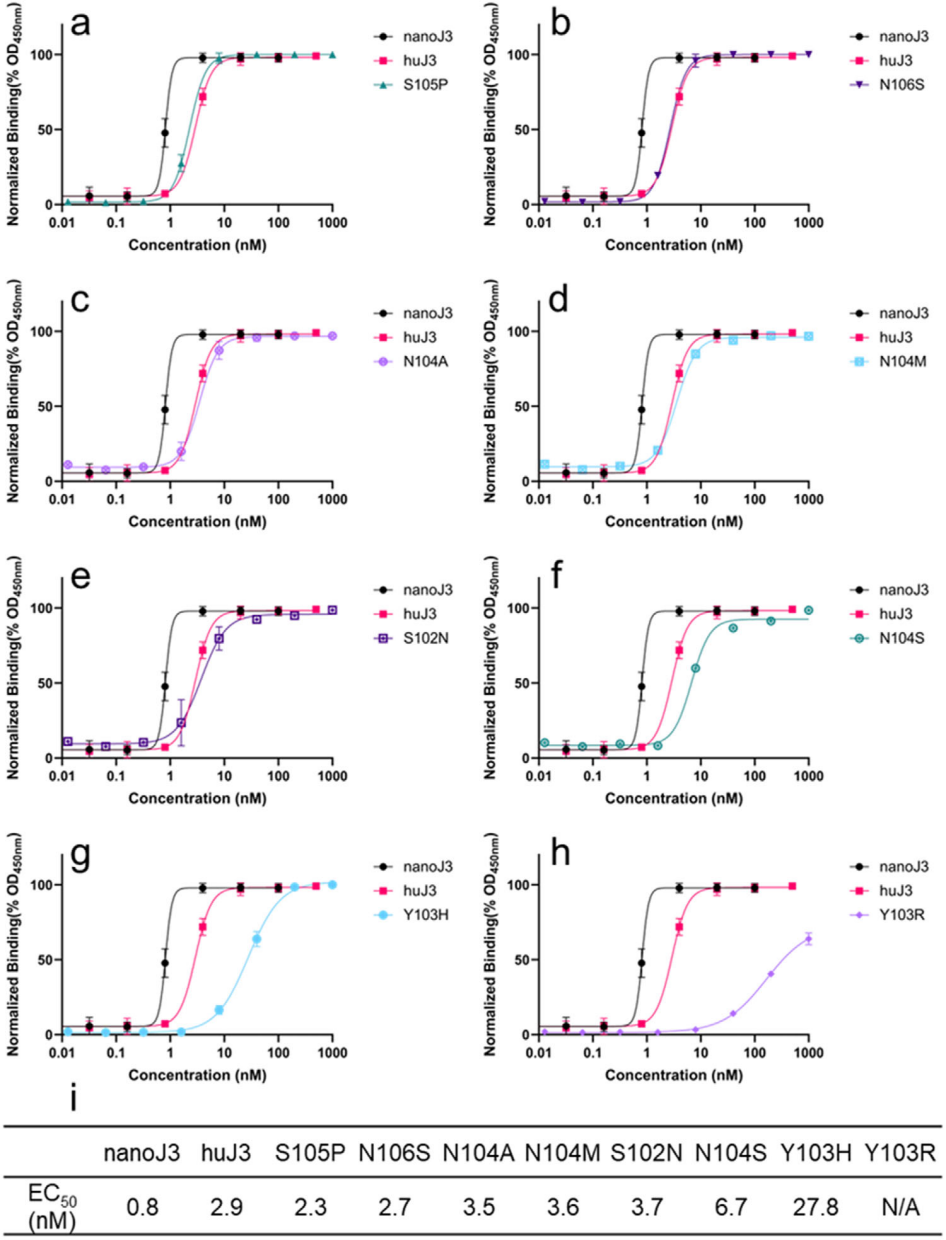
The ELISA binding affinity results for each single‐point design mutation. huJ3 is a humanized version of the llama‐derived nanobody J3, originally designed to target the HIV envelope glycoprotein gp120. a) The binding curve for the single‐point design of S105P. b) The binding curve for the single‐point design of N106S. c) The binding curve for the single‐point design of N104A. d) The binding curve for the single‐point design of N104M. e) The binding curve for the single‐point design of S102N. f) The binding curve for the single‐point design of N104S. g) The binding curve for the single‐point design of Y103H. h) The binding curve for the single‐point design of Y103R. For Figure (a–h), nanoJ3 and huJ3 are used as controls to investigate the binding affinity of each single‐point design. Backbone noise levels (BN) and temperature (T) are set to 0.5 and 0.1, respectively. i) EC_50_ for each single‐point design candidate.

In this work, we investigated the “SYNSN” motif (residues 102 to 106) to conduct stable single‐point antibody sequence designs with experimental validation. We selected design positions at residues 102, 103, 104, 105, and 106, analyzing the most frequent changes. The predictions from AntiBMPNN showed that the most frequent changes for these five positions are S102S (99.0% prefer not to change), Y103R (90.2%), N104S (99.7%), S105P (98.4%), and N106S (>99.9%). Additional designs, such as S102N (<1%), N104A (<1%), N104M (<1%), and Y103H (<1%), were also explored in the present work, as shown in **Figure** **3**. Our findings demonstrated that six out of the eight (accuracy = 75%) single‐point designs resulted in stable J3 sequences with comparable binding affinity to the parental huJ3, including S105P (EC_50_:2.3 nm), N106S (EC_50_:2.7 n), N104A (EC_50_:3.5 nm), N104M (EC_50_:3.6 nm), S102N (EC_50_:3.7 nm), and N104S (EC_50_:6.7 nm). However, Y103H (EC_50_:27.8 nm) led to a 13‐fold decrease in binding affinity, while Y103R caused almost complete loss of binding compared to huJ3 (EC_50_:2.9 nm). These computational and experimental results further support the robustness of AntiBMPNN. To further assess the feasibility of AntiBMPNN, we extended the sequence design to the complementarity‐determining regions (CDRs) of antibodies or nanobodies.

### 2.6 Sequence Design of the Full‐Length of huJ3 CDR1 Using AntiBMPNN with Experimental Validation

Median backbone noise level (BN = 0.5) and temperature (*T* = 0.1) were employed to design the full‐length of the CDR1 region of huJ3 (motif: “GSIFNQYA,” residues 26 to 33) and generate the complete sequences. The results revealed that positions 26 (“G”), 27 (“S”), 29 (“F”), 32 (“Y”), and 33 (“A”) were highly conserved, even as the BN increased to 0.7. This suggests that increasing BN is necessary to capture variability in these positions. In contrast, positions 28 (“I”), 30 (“N”), and 31 (“Q”) exhibited higher variability, changing to other residues.

**Figure 4:**
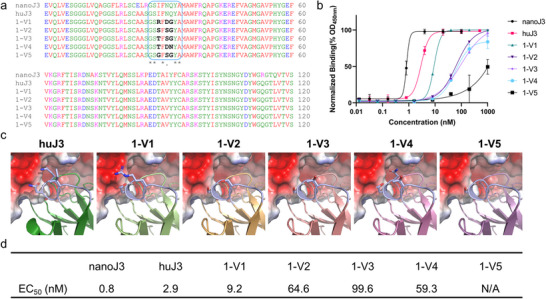
Experimental validation results for the AntiBMPNN‐designed huJ3 CDR1. a) Comparison of sequences for each variant (1‐V1 to 1‐V5), with differing residues highlighted in black. b) Binding curves for each variant (1‐V1 to 1‐V5), with distinct lines representing individual variants to show variations in binding affinities. c) 3D structural complexes of the gp120 and designed huJ3, with the CDR1 regions of each variant depicted as cartoon diagrams. Changed positions are shown as stick representations, while the gp120 protein is rendered as a surface structure, color‐coded by electrostatic potential. d) EC_50_ values for each variant (1‐V1 to 1‐V5).

The top five sequences, 1‐V1 to 1‐V5, identified by the scoring function and AlphaFold 3 (Table S1, Supporting Information), were selected for experimental validation. **Figure** **4**a illustrates the alignment of these sequences with two controls, nanoJ3, and huJ3, focusing on the CDR1 region. Residues differing from the controls are highlighted in black. Figure 4b presents the results of ELISA assays for each variant, with nanoJ3 and huJ3 serving as positive controls. Figure 4c provides a comparison of the binding poses between these huJ3 variants and gp120, highlighting the interactions and contributions of the designed residues relative to those in the original huJ3. Among the variants, Q31 was primarily replaced by glycine, which has a relatively small side chain volume, leading to insufficient contact at this position. Variant 1‐V1 compensates with an I28R mutation, utilizing the longer side chain of arginine. However, other variants lack this compensatory mechanism, resulting in a noticeable decrease in their binding affinity. Figure 4d displays the EC_50_ values for all sequences. All experimentally validated sequence designs were computationally screened and the ID is sorted according to affinity.

From Figure 4, the EC_50_ values for the positive controls, nanoJ3, and huJ3, are 0.8 and 2.9 nm, respectively. Among the designed variants, 1‐V1 to 1‐V4 exhibited EC_50_ values ranging from 9.2 to 99.6 nm, while the binding activity of 1‐V5 was not detected. Notably, Figure 4c reveals that designed residues R28 and D30 in 1‐V1 made significant contributions to binding with the gp120 surface, resulting in better binding affinity compared to other variants. Typically, the antibody CDR1 region may be less critical for antigen binding than CDR3. In the following section, we applied AntiBMPNN to design the CDR3 region of huJ3 with experimental validations.

### 2.7 Sequence Design of huJ3 CDR3 (Residues 94 to 105) Using AntiBMPNN with Experimental Validation

In addition to CDR1, we also conducted design within the CDR3 motif of huJ3, focusing specifically on residues 97 to 106, which are part of the full CDR3 sequence “ARSKSTYISYNSNGYDY” (residues 94–110). In the original study of the J3 antibody, it was reported that single‐point mutations—T98R and Y99G (corresponding to T99 and Y100 in our numbering)—within the conserved “STY” motif (residues 98–100) led to a complete loss of binding activity. This highlights the functional sensitivity of this motif and the challenges involved in designing viable variants.

Utilizing the same huJ3–gp120 complex and design parameters (BN = 0.5, *T* = 0.1), we focused our design efforts specifically on residues 94 to 105, which comprise a portion of the full CDR3 sequence “ARSKSTYISYNSNGYDY” (residues 94–110). During the design process, the critical “STY” motif (residues 98–100) was fixed to preserve binding functionality. Our results indicated that residues at positions 95 (“R”), 97 (“K”), 102 (“S”), 104 (“N”), 105 (“S”), and 106 (“N”) were frequently substituted with other residues. The eleven most frequent sequences, ranked by the scoring function and AlphaFold 3, were selected for experimental validation.

**Figure 5:**
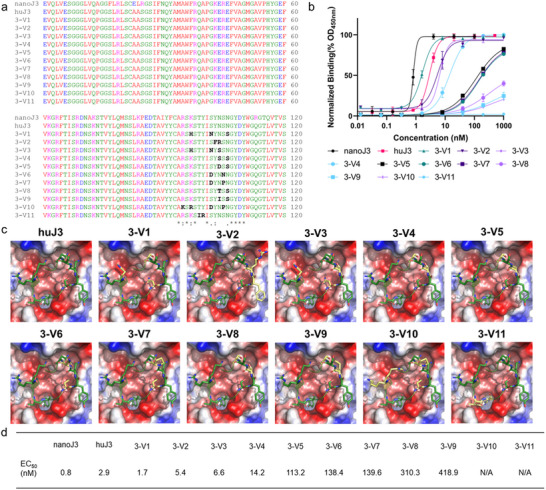
Experimental validation outcomes for the AntiBMPNN‐designed huJ3 CDR3 variants. a) Comparison of the sequences for each variant (3‐V1 to 3‐V11), with residues differing between variants highlighted in black. b) Binding curves for each variant (3‐V1 to 3‐V11), with distinct lines representing individual variants to illustrate variations in their binding affinities. c) 3D structural models of the designed huJ3 complexed with gp120, with the CDR3 regions of each variant depicted as cartoon diagrams. The altered residues are shown as stick representations, and the gp120 protein is rendered as a surface structure, color‐coded by electrostatic potential. d) EC_50_ values for each variant (3‐V1 to 3‐V11).

The EC_50_ values for the re‐engineered variants 3‐V1 through 3‐V9 varied widely, ranging from 1.7 to 418.9 nm. Importantly, the EC_50_ value for 3‐V1 (1.7 nm) was better than that of huJ3 (2.9 nm), due to the significant contributions of three designed residues in CDR3‐H97, N102, and S106‐to binding with gp120 (**Figure** **5**c). Similarly, the designed residues in 3‐V3, which include H97, N102, and S104, resulted in an EC_50_ of 6.6 nm, slightly lower than that of huJ3 (Figure 5b,c). Additionally, the EC_50_ values for 3‐V10 and 3‐V11 were “non‐binding” (N/A), which is attributed to inappropriate modifications at crucial positions such as R95K, T99I, and Y100R. These designs further validated the efficiency of AntiBMPNN.

### 2.8 Sequence Design of CDR2 of an Anti‐CD16A VH antibody (D6) Using AntiBMPNN with Experimental Validation

To further assess the robustness and feasibility of AntiBMPNN, we applied it to design the CDR2 region of D6, a single domain antibody (the human heavy chain variable domain VH) that targets human CD16A for NK engagers with therapeutic applications in cancer immunotherapy and the treatment of various hematologic malignancies.^[^ ^27^ ^]^

We first constructed the 3D structure of D6 as described in the Experimental Section. Using this 3D model and the same parameters (BN = 0.5, *T* = 0.1), we focused on designing the CDR2 region, which consists of the motif “SIYYSGSTN” (residues 50 to 58). Our results indicated that residues at positions 50 (“S”), 51 (“I”), 53 (“Y”), 54 (“S”), and 56 (“S”) were more easily substituted with other residues compared to positions 52 (“Y”), 55 (“G”), 57 (“T”), and 58 (“N”). Additionally, since the R66L mutation in the original D6 antibody maintained binding affinity, we combined the R66L mutation with the CDR2 design of D6.

**Figure 6:**
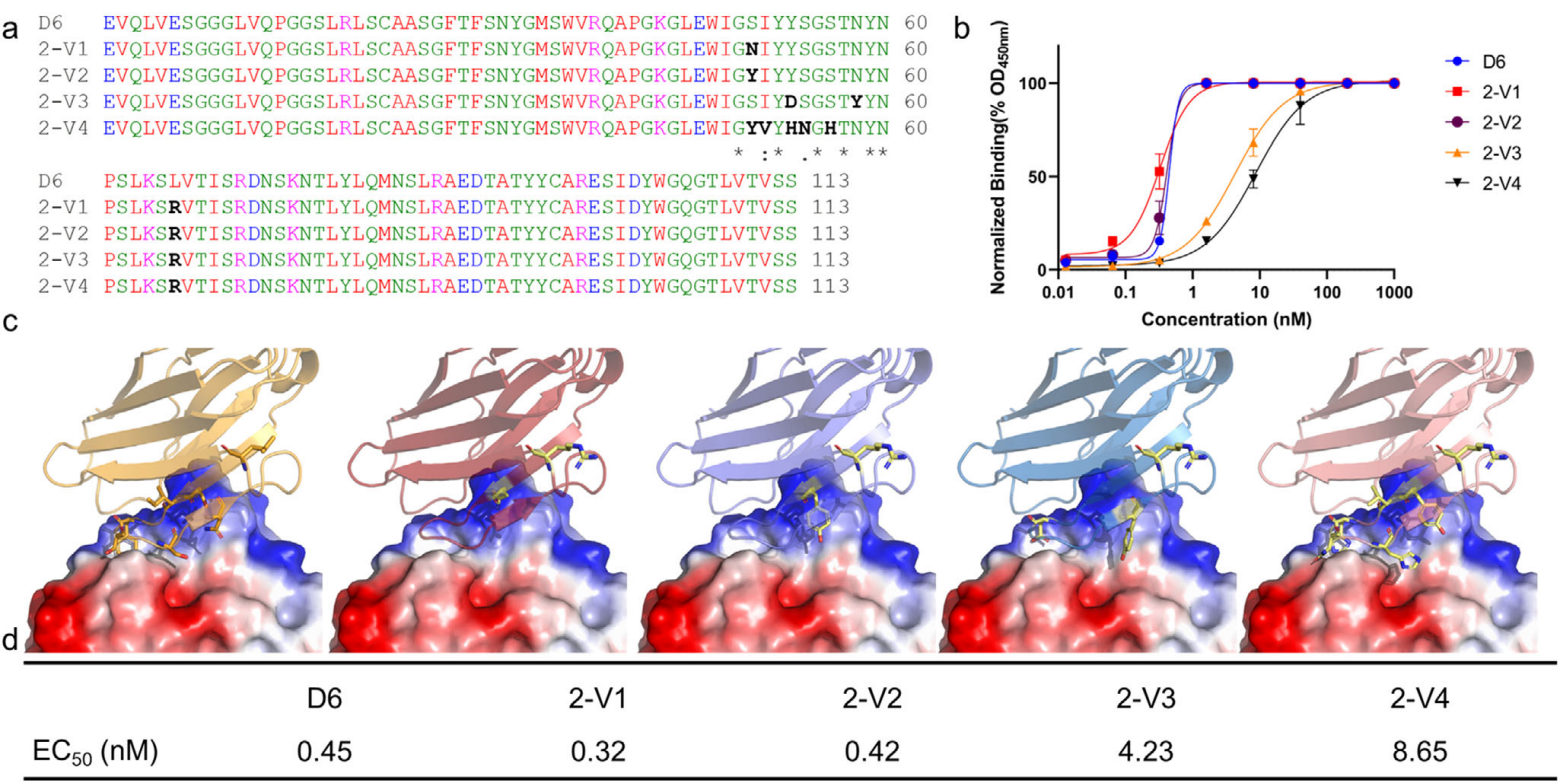
Experimental validation outcomes for the AntiBMPNN‐designed D6 CDR2 variants. a) Comparison of the sequences for each variant (2‐V1 to 2‐V4), with residues differing between variants highlighted in black. b) Binding curves for each variant (2‐V1 to 2‐V4), with distinct lines representing individual variants to illustrate variations in their binding affinities. c) 3D structural models of the designed D6 complexed with CD16A, with the CDR2 regions of each variant depicted as cartoon diagrams. Altered residues are shown as yellow stick representations, and the CD16 protein is rendered as a surface structure, color‐coded by electrostatic potential. d) EC_50_ values for each variant (2‐V1 to 2‐V4).

The top four sequences—2‐V1, 2‐V2, 2‐V3, and 2‐V4—identified by the scoring function, were selected for experimental validation (**Figure** **6**). Figure 6a shows the sequence alignment of these variants with D6 as the control, with differences in residues highlighted in black. Figure 6b presents ELISA assay results for each variant, with D6 serving as the positive control. Figure 6c compares the binding poses of these nanobodies with CD16, highlighting the contributions of the designed residues relative to the original D6. According to our docking results, the tyrosine at position 53 is clearly in a crucial position. Its side chain phenol group forms a spatially complementary structure with the local surface of the antigen. Variants containing a mutation at this site (2‐V1 and 2‐V4) demonstrated an obvious decrease in affinity. In contrast, position S50 appears to be more tolerant; a slight extension of the side chain resulted in increased affinity (2‐V2), and even a larger side chain showed no negative impact (2‐V3). Figure 6d illustrates the EC_50_ values for all sequences.

According to Figure 6, the EC_50_ value of the positive control D6 is 0.4 nm. The EC_50_ values for 2‐V1, 2‐V2, 2‐V3, and 2‐V4 are 0.3, 0.4, 4.2, and 8.6 nm, respectively. Notably, Figure 6d reveals that 2‐V1 and 2‐V2 exhibited stronger binding affinity compared to the other two variants, with both 2‐V1 (S50N) and 2‐V2 (S50Y) sharing the design at the same position of “S”(50). The detailed interactions between these variants and CD16 are shown in Figure 6c.

### 2.9 Comparative Analysis of AntiBMPNN with Existing Methods for Antibody Sequence Design Based on Experimental Data

To evaluate the performance of different generative models, we first tasked AntiBMPNN, ProteinMPNN, and AbMPNN with redesigning residues 97 to 106 of the CDR3 region, while fixing the critical “STY” motif. These models proposed variants with 2 to 5 mutations; however, none of the designed sequences retained measurable binding activity (see Figure S3, Supporting Information). This outcome reinforces the importance of maintaining key functional residues and carefully managing mutation scope to allow meaningful comparison across design approaches.

**Figure 7:**
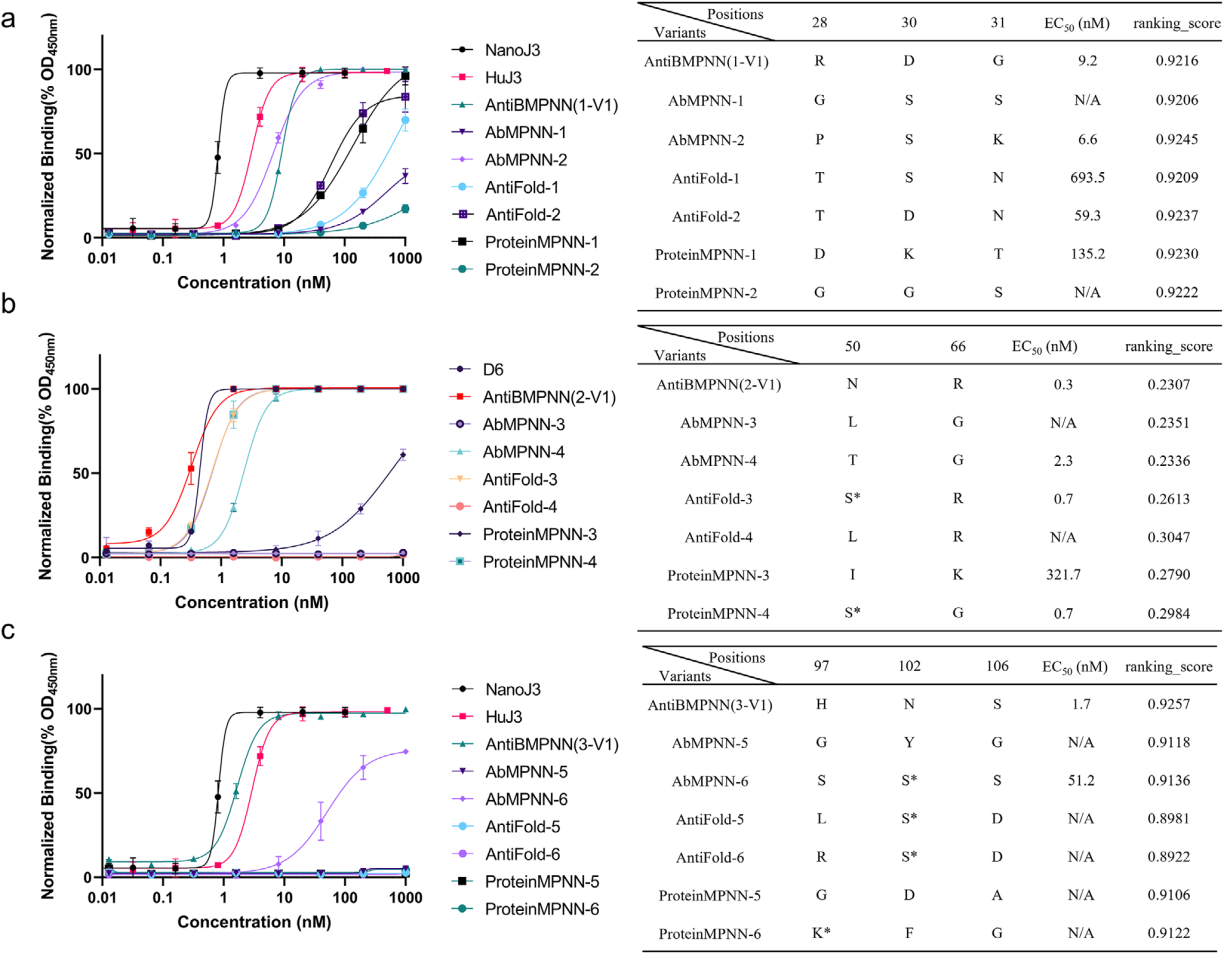
Comparison of AntiBMPNN with existing methods for antibody sequence design based on experimental data. In the huJ3 project, a) AntiBMPNN optimized CDR1, achieving a EC_50_ of 9.2 nm, outperforming ProteinMPNN (135.2 nm) and AntiFold (59.3 nm). While it showed stronger performance than these methods, it displayed comparable capability to AbMPNN, which achieved a EC_50_ of 6.6 nm in this case. b) AntiBMPNN optimized the D6 CDR2 region, achieving a EC50 of 0.3 nm, outperforming AbMPNN (2.3 nm), AntiFold (0.7 nm), and ProteinMPNN (0.7 nm). c) AntiBMPNN optimized the CDR3 region of huJ3, achieving a EC_50_ of 1.7 nm, outperforming AbMPNN (51.2 nm), AntiFold (non‐binding), and ProteinMPNN (non‐binding). The numbers in the table represent the residue positions. “*” indicates that the residue at the corresponding position was not altered by the algorithms. The “ranking_score” was generated by AlphaFold 3 for all the designed sequences complexed with their antigen.

We presented a comprehensive comparison of AntiBMPNN with established approaches, including AbMPNN, AntiFold, and ProteinMPNN, as shown in **Figure** **7**, with identical large‐scale sampling across models and conducting experimental tests based on the built‐in scoring functions and AF3 modeling screening.

For the huJ3 project, we focused on optimizing the CDR1 region, specifically residues 28, 30, and 31, which played a crucial role in huJ3's binding affinity to gp120. AntiBMPNN designed the sequence “R” (position 28), “D” (position 30), and “G” (position 31), achieving an impressive EC_50_ of 9.2 nm. In comparison, AbMPNN produced two designs: “GSS” and “PSK” at positions 28/30/31, with EC_50_ values of N/A(non‐binding) and 6.6 nm (Figure 7), respectively. The poor performance of “GSS” demonstrated AbMPNN's limitations in generating high‐affinity sequences for this region in some cases. AntiFold's best design, “TDN” is the same as 1‐V4 designed by AntiMPNN, resulted in a EC_50_ of 59.3 nm, indicating suboptimal binding affinity and reduced effectiveness in optimizing the CDR1 region. ProteinMPNN generated the designs “DKT” and “GGS” for residues 28/30/31, with EC_50_ values of 135.2 nm and N/A(non‐binding), respectively, both of which were significantly outperformed by AntiBMPNN. In conclusion, AntiBMPNN demonstrated strong performance in optimizing binding affinity for CDR1 in the huJ3 project, achieving a EC_50_ of 9.2 nm. While outperforming AbMPNN and ProteinMPNN, AntiBMPNN fell short of AntiFold in some cases, indicating strong but not universally superior performance.

For the D6 antibody (Figure 7), we targeted the CDR2 region, specifically focusing on residues 50 and 66. AntiBMPNN designed the optimal sequence “NR,” achieving an exceptionally low EC_50_ of 0.3 nm, demonstrating the method's precision in enhancing binding potential. AbMPNN's top designs, “LG” and “TG,” yielded mixed results: “LG” cannot be expressed by E. coli, while “TG” had a EC_50_ of 2.3 nm. Although moderately successful, “TG” still did not match the superior binding of AntiBMPNN's “NR.” AntiFold's best design, “SR,” retained the residue at position 50 and achieved a of 0.7 nm, which, while competitive, still fell short of AntiBMPNN's result. Meanwhile, its “LR” design yielded no measurable binding (N/A). ProteinMPNN's designs, “SK” and “SG,” performed poorly, with “SK” showing no detectable binding (EC_50_ > 1000 nm) and “SG” reaching a EC_50_ of 0.7 nm. In summary, AntiBMPNN outperformed all other methods for CDR2, highlighting its robustness in designing high‐affinity sequences.

In the CDR3 region of huJ3 (Figure 7), targeting residues 97, 102, and 106, AntiBMPNN again demonstrated its superior performance. The optimal sequence “HNS” achieved a EC_50_ of 1.7 nm, illustrating the method's effectiveness in enhancing binding interactions. AbMPNN's top designs, “GYG” (EC_50_: N/A, non‐binding) and “SSS” (EC_50_ of 51.2 nm), underperformed, with “SSS” retaining the residue at position 102. The lack of binding data for “GYG” indicates challenges in designing effective sequences in this region. AntiFold's designs, “LSD” and “RSD” also retained position 102 and had no measurable EC_50_, showing its limited effectiveness. ProteinMPNN's designs, “GDA” and “KFG,” failed to produce detectable binding, further underscoring the method's difficulties in optimizing CDR3. AntiBMPNN's “HNS” sequence demonstrated clear improvements in binding affinity, outperforming designs from AbMPNN, AntiFold, and ProteinMPNN.

We applied AlphaFold 3 to generate the “ranking_score” for all the designed sequences complexed with their antigen, as listed in Figure 7. Although AlphaFold 3 derived ranking_scores provide useful estimates of structural plausibility, we observed that these scores did not always correlate well with experimental binding activity. For instance, in the huJ3 re‐design, two antibodies with nearly identical ranking_scores (9.245 and 9.230) showed over an order of magnitude difference in EC₅₀ values (6.6 nm vs 135.2 nm). This suggests that structural confidence alone does not fully capture the functional consequences of CDR variation. Furthermore, the D6 designs consistently showed lower ranking_scores (≈0.2) compared to huJ3 (≈0.9), which may be attributed to the lack of experimentally resolved structures for D6 and its antigen. In the absence of crystal or cryo‐EM structures, AlphaFold 3 predictions for D6‐based designs may be less accurate, resulting in lower structural confidence scores. These findings underscore the limitations of relying solely on AlphaFold 3 ranking_scores for functional prediction and emphasize the need to integrate other metrics or experimental validation.

## 3 Discussion

Antibody engineering has traditionally relied upon expert‐driven design combined with extensive in vitro and in vivo experimental screening. Recently, various computational methods for antibody design have been developed. An obvious limitation of many existing computational algorithms is that their effectiveness is predominantly demonstrated at the computational level without sufficient experimental validation to confirm real‐world applicability. ProteinMPNN represents the first experimentally validated deep learning model for protein sequence design, and its success in multiple protein design tasks underscores a significant advancement in the field. Importantly, this indicates that the MPNN architecture, derived from the transformer model, holds promise as an effective model for protein design.

Regarding dataset selection, we deliberately focused solely on experimentally derived structural data to enhance the reliability of the model's predictions. However, this approach inherently limits the volume of available training data. To address this limitation, we removed redundant sequences and separated antibody heavy and light chains into individual domains, thus forming domain‐specific antibody datasets. This approach contrasts sharply with methodologies employed by AbMPNN and AntiFold models, which utilize dominantly modeled structural datasets, potentially introducing systematic biases. Figure 1e shows the average sequence recovery rates for all six CDR loops (CDRH1‐3 and CDRL1‐3) on our antibody test set. We compared four models‐AntiBMPNN, AntiFold, AbMPNN, and ProteinMPNN. AntiBMPNN performed best overall, with the highest recovery rates in four out of six CDRs, including the important CDRH3, CDRL1, and CDRL2 regions. While AntiFold performed competitively on CDRL3 and CDRH2, both AbMPNN and ProteinMPNN showed lower recovery rates across most regions. These results underscore the robustness of AntiBMPNN and its enhanced capacity to generate native‐like CDR sequences when assessed on a carefully curated and representative testing set.

Another critical aspect involves the evaluation of candidate sequences once generated. Traditional methods often rely on structure modeling tools like AlphaFold2 for scoring. But these methods depend heavily on model accuracy and the completeness of template databases, which can lead to poor results when structural templates are missing. We also tested different models using both default settings and large‐scale sequence generation followed by clustering. We found that most sequences from the default setting also appeared among the high‐frequency sequences in the large‐scale designs. However, the large‐scale approach additionally produced many new and frequently recurring sequences. This highlights the value of generating more sequences and applying clustering, as it not only provides a new metric for evaluating the generated sequences but also helps identify candidates that are theoretically closer to the training data and therefore more likely to represent viable antibodies. Notably, other models did not incorporate further optimization at this stage. In contrast, our approach leverages these additional steps to enhance candidate quality. Finally, the modeling results from AlphaFold3 can be integrated to comprehensively evaluate the selected sequences.

Nevertheless, several methodological aspects require further improvement to enhance our model's applicability. A primary goal for future studies is integrating antigen structural information directly into antibody sequence design when antigen structures are available. Additionally, incorporating molecular dynamics simulations during sequence screening may allow for dynamic evaluation and more effective selection, particularly in scenarios lacking adequate modeling templates.

Moreover, future work will also include the co‐design of multiple amino acid residues across different complementarity‐determining regions (CDRs) to robustly assess broader model performance. Another significant research direction involves improving model capabilities for designing antibody framework regions, thereby potentially expanding the applicability and effectiveness of antibody engineering efforts.

## 4 Conclusion

This study presents AntiBMPNN, a deep‐learning framework designed to enhance antibody sequence optimization. By utilizing a specialized antibody 3D dataset, a finely tuned Message Passing Neural Network (MPNN), a frequency‐based scoring function, and AlphaFold 3, AntiBMPNN enables highly precise antibody sequence design. AntiBMPNN demonstrates superior performance compared to ProteinMPNN, with reduced perplexity (1.5) and improved sequence recovery (≥80%). Experimental results show a 75% success rate in single‐point antibody design. In most cases, AntiBMPNN outperforms established methods, including AbMPNN, AntiFold, and ProteinMPNN, producing better binding affinities in the design of complementarity‐determining regions (CDRs) CDR1, CDR2, and CDR3. In the NanoJ3 project, AntiBMPNN optimized CDR1, achieving a EC_50_ of 9.2 nm, outperforming ProteinMPNN (135.2 nm) and AntiFold (59.3 nm). While its performance surpassed these methods, it showed comparable results to AbMPNN, which achieved a EC_50_ of 6.6 nm. For the D6 CDR2 region, AntiBMPNN achieved a EC_50_ of 0.3 nm, outperforming AbMPNN (2.3 nm), AntiFold (0.7 nm), and ProteinMPNN (0.7 nm). In the CDR3 region of J3, AntiBMPNN achieved a EC_50_ of 1.7 nm, outperforming AbMPNN (51.2 nm) and demonstrating superior performance compared to AntiFold and ProteinMPNN, both of which showed no detectable binding. Additionally, All the modeling results of antibodies and complexes of antibody and antigen by AlphaFold 3 can be found in Table S2 (Supporting Information). These results highlight AntiBMPNN's ability to design sequences with superior binding affinities, demonstrating the utility of our approach in supporting antibody sequence optimization workflows.

## 5 Experimental Section

### Training Datasets

X‐ray crystallographic and cryo‐electron microscopy (cryo‐EM) antibodies and/or antibody–antigen complexes sourced from the Structural Antibody Database^[^ ^28^ ^]^ (SAbDab: https://opig.stats.ox.ac.uk/webapps/sabdab‐sabpred/sabdab), deposited prior to January 22nd, 2024, were utilized, employing a resolution cutoff of 5 Å. A total of 8078 antibody structures dataset was obtained. To construct a single‐domain antibody dataset, paired antibodies were separated into independent heavy chain (H) and light chain (L) domain structures. Thus, each chain from paired antibodies was treated as an individual entry, effectively enhancing dataset size and diversity. This approach mitigated limitations caused by the relatively limited number of available high‐resolution antibody structures, contributing to improved model robustness without compromising data quality.

To minimize redundancy and ensure functional diversity, a two‐stage deduplication procedure was implemented. Initially, exact sequence duplicates and redundant structural entries were removed. Following this, the remaining antibody sequences underwent clustering based on 30% sequence identity within their complementarity‐determining regions (CDRs). This clustering step significantly reduced redundancy related to the functional similarities of antibodies. No structures containing multiple copies of the antibody were included in the training set. Instead, they were randomly sampled during training to ensure the integrity and diversity of the dataset.

For the test cluster, the currently released structures were downloaded from SAbDab that were deposited after January 22nd, 2024. For the test cluster, the data were cleaned up using the same protocol as described previously. This resulted in a test set of crystal/cryo‐EM structures consisting of 40 H+L‐chain, 40 H‐chains, and 40 L‐chain, totaling 120 3D structures.

### AntiBMPNN Architecture

The AntiBMPNN model was built upon structured transformers,^[^ ^29^, ^30^, ^31^ ^]^ leveraging a message‐passing neural network (MPNN)^[^ ^25^ ^]^ as the aggregation function.

### Graph Representation (Encoder Processing)

Antibody structures were represented as graphs, with atoms as nodes and atomic interactions (e.g., bonds, distances) as edges. Each node had a feature vector capturing atomic properties such as type, coordinates, charge, and hydrophobicity. Each edge encoded pairwise interactions, including bond type and distances, potentially using radial basis function (RBF) values. This representation transformed the input structure into a graph format for neural network processing.

### Message Passing Layers (Encoder Processing)

AntiBMPNN used MPNN layers to propagate information between nodes (atoms), iteratively updating their representations based on interactions with neighbors.

(1) $$h_{v}^{\left(l+1\right)}=\sigma \left(W\;\times \;h_{v}^{\left(l\right)}+\sum_{u\in N\left(v\right)}W_{euv}\;\times \;h_{u}^{\left(l\right)}\right)$$

where $h_{v}^{\left(l\right)}$ / $h_{u}^{\left(l\right)}$ is the hidden state of node *v* / *u* at layer *l*, σ is an activation function, *W* is a learnable weight matrix, and *W_euv_* is a learnable edge‐specific weight matrix. Typically, multiple message‐passing layers were utilized to capture hierarchical and spatial features of the antibody structure.

### Node and Edge Update Functions (Decoder Processing)

In each layer, node, and edge update functions computed new representations based on aggregated information from neighboring nodes:

(2) $$h_{v}^{\left(l+1\right)}=f_{node}\;\left(h_{v}^{\left(l\right)},\sum_{u\in N\left(v\right)}W_{euv}\;\times \;h_{u}^{\left(l\right)}\right)$$

where *f_node_* is a learnable node update function.

Edge update functions refine edge representations, capturing spatial relationships and interactions within the antibody:

(3) $$e_{uv}^{\left(l+1\right)}=f_{edge}\;\left(e_{uv}^{\left(l\right)},h_{v}^{\left(l\right)},h_{u}^{\left(l\right)}\right)$$

where *f_edge_* is the edge update function, $e_{uv}^{\left(l\right)}$ represents the updated feature of the edge (*u*, *v*) after layer *l*, $h_{v}^{\left(l\right)}$ is the feature of node *v* at layer *l*, $h_{u}^{\left(l\right)}$ is the feature of node *u* at layer *l*.

### Graph Pooling (Decoder Processing)

After several message‐passing layers, AntiBMPNN used graph pooling to aggregate node representations into a global antibody structure.

(4) $$z=\sum_{v\in V}W_{pool}\;\times \;h_{v}^{\left(l\right)}$$

where *z* is the pooled representation, *W_pool_* is a learnable weight matrix, and *l* is the number of message‐passing layers.

### Training and Optimization (Decoder Processing)

AntiBMPNN was trained using supervised learning on antibody datasets, minimizing a categorical cross‐entropy loss to reduce the difference between the predicted and actual amino acid sequences:

(5) $$L=-\sum_{i}\sum_{c=1}^{20}y_{i,c}\log\left(p_{i,c}\right)$$

where *p*_*i*,*c*_ is the predicted probability of amino acid class *c* at position *i*, *y*_*i*,*c*_ is the one‐hot encoded true label for the amino acid at position *i*.

Optimization methods such as Adam optimizer were used to update the model parameters and improve performance:

(6) $$\theta =\theta \;-\;\alpha \nabla_{\theta }L\left(\theta \right)$$

where θ is the set of learnable parameters, α is the learning rate, and ∇_θ_ *L*(θ) is the gradient of the loss function with respect to the parameters.

This approach enables the model to effectively predict amino acid sequences conditioned on structural context, including fixed regions such as antibody frameworks.

### Sequence Generation (Decoder Processing)

AntiBMPNN predicted the amino acid sequence from 3D structures using an autoregressive approach, linking structure to sequence:

(7) $$p\left(x|s\right)=\prod_{i}p\left(x_{i}|s,\;x_{<i}\right)$$

here, *p*(*x_i_* ∣*s*, *x*_<*i*_) denoted the conditional probability of the amino acid *x_i_* at decoding step *i*, and $x_{<i}=\{x_{1\;}\;,\text{…},x_{i-1}$} referred to previously decoded residues. The probabilities were parameterized using an encoder for node and edge embeddings and a decoder that predicted the next residue autoregressively from prior residues and structural embeddings.

### Scoring Function for Selecting Candidate Sequences

The algorithm generated 32 768 candidate antibody sequences, which were analyzed and ranked based on their similarity to the reference sequence using a frequency‐based scoring function. The number 327 68 (2¹⁵) was chosen primarily because it is large enough to demonstrate the model's ability to generate a diverse set of candidate sequences for a specific design task. The input reference sequence served as a benchmark for comparison, with the algorithm focusing on specific segments (designed positions) for analysis. For each position in the candidate sequences, the Hamming distance was calculated between the amino acids of the candidate sequence and the reference sequence:

(8) $$D_{i,j}=\;\mathrm{Hamming}\left(x_{ij},x_{j}^{\mathrm{ref}}\right)$$

*D*_*i*,*j*_ is the difference score between the *j*‐th position of sequence *i* and the reference sequence; Hamming(*a*, *b*) is the Hamming distance between amino acids *a* and *b*.

Candidate sequences were then aggregated based on sequence recovery metrics, grouping similar sequences and identifying recurring patterns or variants. The differences between each candidate sequence and the reference sequence were recorded as:

(9) $$\Delta_{ij}=x_{ij}\;-\;x_{j}^{ref}$$

Δ_*ij*_ represents the change at position *j* of sequence *i*, *x_ij_* is the amino acid in sequence *i* at position *j*, and $x_{j}^{ref}$ is the amino acid in the reference sequence at position *j*.

A frequency‐based scoring function was applied to assess the significance of sequence modifications:

(10) $$F_{i}=\frac{Frequency\;of\;x_{i}}{Total\;occurences}$$

*F_i_* is the frequency score for the sequence *i*.This scoring function analyzed the frequency and significance of changes within candidate sequences relative to the reference sequence.

All sequences were clustered, and each cluster was ranked by frequency, with higher‐ranking clusters indicating more promising modifications. Physical descriptors,^[^ ^32^ ^]^ such as residue volume, hydrophilicity, and charge at pH 7.4, were also incorporated to refine the selection of sequences. The top‐ranked sequences were selected for further investigation by AlphaFold 3.

The algorithm's output included a detailed CSV file containing ranked sequences, metadata on sequence changes relative to the reference, and information on the frequency and nature of modifications, providing valuable insights into the optimization potential for antibody design.

### AntiBMPNN Model Inference

During inference, the model required the backbone structural coordinates as essential input, since the model relied on spatial information to predict amino acid sequences. It constructed a locally connected graph where each residue was influenced by its 48 nearest Cα neighbors, reflecting the observation that local backbone geometry largely determined residue identity. The choice of 48 nearest neighbors was based on findings from the ProteinMPNN paper, where sequence recovery performance was shown to saturate at this value; this model similarly adopted this setting to balance efficiency and accuracy. Additionally, AntiBMPNN incorporated partial sequence context, where known residues, such as conserved regions in antibodies, were provided as fixed inputs to guide the design of variable regions. While masking sequence regions had limited impact because the model was designed to operate with unknown sequences, masking structural coordinates, especially within a residue's local neighborhood, led to a significant drop in performance. This was because accurate 3D context was critical for determining valid amino acid choices. Therefore, providing complete structural information and, when applicable, fixed sequence regions enhanced inference accuracy and ensured biologically relevant designs.

The antibody structures huJ3 and D6 utilized in this study were sourced from prior publications.^[^ ^26^, ^33^ ^]^

### 3D Structural Modeling of nanoJ3, huJ3, and the D6 Nanobody Complexed with Antigens Using AlphaFold 3 and ClusPro and Rosetta

AlphaFold 3^[^ ^34^ ^]^ was cloned from the official repository (https://github.com/google‐deepmind/alphafold3) and was installed locally following the official installation documentation. The model parameters were required and received from Google. AlphaFold 3 no longer required specifying monomer or multimer mode, so only one sequence was input for modeling, which could be considered as monomer modeling. In Alphafold3, the metrics used to evaluate the quality of monomer modeling was the atomic‐level pLDDT (predicted Local Distance Difference Test), a per‐atom confidence estimate on a 0–100 scale where a higher value indicated higher confidence. The average pLDDT of each structure was the average of each atom. For multimer modeling, it was only needed to add additional chains to the JSON file to perform multimer modeling. The “ranking_score” in AlphaFold 3 was a composite metric used to rank predicted multimer models based on structural confidence, interface reliability, disorder proportion, and atomic clash status. It was calculated using the equation: ranking_score = 0.8 × ipTM + 0.2 × pTM + 0.5 × disorder − 100 × has_clash, where ipTM (interface predicted TM‐score) measured confidence in inter‐chain interfaces, and pTM (predicted TM‐score) indicated the overall structural confidence, both ranging from 0 to 1. The disorder term, also ranging from 0 to 1, represented the fraction of predicted disordered residues in the structure. The “has_clash” term was a Boolean value signaling whether significant atomic clashes occur, becoming true if more than 50% of atoms or over 100 atoms were involved in clashes. With a scoring range from −100 to 1.5, higher ranking_score values corresponded to more reliable structural predictions. Bash scripts for running AlphaFold3 were uploaded to the AntiBMPN's GitHub repository.

The crystal structure of llama VHH nanobody J3 (nanoJ3) was retrieved from its complex with gp120 CladeC (PDB: 7ri1)^[^ ^35^ ^]^ from the Protein Data Bank^[^ ^36^ ^]^ (https://www.rcsb.org/). For huJ3, D6, and other designed sequences, both monomer and multimer modeling, including their corresponding antigens were performed. Five predictions were generated for each modeling task, and the model with the highest pLDDT or ranking_score was selected as the final result, summarized in Table S1 (Supporting Information).

For the huJ3 variants, consistent modeling behavior was observed, with all antibodies docking at the same position. In contrast, the D6 variants were modeled differently, likely due to the absence of an anti‐CD16a antibody–antigen complex in the PDB. To address this, structure‐based protein–protein docking methods were employed to predict potential binding complexes, as previously described.^[^ ^26^ ^]^ The antibody and antigen were first submitted to the ClusPro^[^ ^37^ ^]^ server to obtain the global docking complex. Subsequently, the “docking_protocol” from Rosetta^[^ ^19^ ^]^ was employed for local refinement. Finally, the complexes that met the criteria were selected as the final results and were visualized via PyMol, including cartoon and stick representations and protein contact potentials.

### Antibody Sequence Design by ProteinMPNN

The design process using ProteinMPNN began by cloning the repository from GitHub (https://github.com/dauparas/ProteinMPNN) and setting up the required conda environment to ensure compatibility with all dependencies. After the environment was successfully set up, the antibody structure files were organized and stored in separate subfolders within the “input” directory of the ProteinMPNN project.

According to the guidelines provided on the GitHub page, the “submit_example_4_non_fixed.sh” script was used as a template for submitting the design job. This script was modified to target specific antibody positions for optimization. The modification involved adjusting the following parameters for the design process:

- Number of Sequences per Target (–num_seq_per_target): This parameter was set to 32 768, meaning that for each target antibody structure, ProteinMPNN would generate 32 768 antibody sequences. This ensured that a variety of potential sequences were considered for each target, increasing the likelihood of finding an optimized design.
- Backbone Noise (–backbone_noise): Set to 0.5, this parameter controlled the noise applied to the antibody backbone during the design process. A value of 0.5 striked a balance between flexibility and structure preservation, allowing for some degree of conformational variation while maintaining the core structural integrity.
- Sampling Temperature (–sampling_temp): Set to 0.1, this parameter controlled the randomness of the sampling process. A low value like 0.1 encouraged the model to explore lower‐energy states and generated more refined, energetically favorable sequences.

Once these modifications were made, the script was executed in the configured conda environment. ProteinMPNN utilized its trained models and optimization algorithms to design sequences that would potentially improve the binding affinity and overall stability of the antibody. The output consisted of newly designed sequences, each associated with a predicted structural representation, which were then saved for further analysis and validation.

### Antibody Sequence Design by AbMPNN

To design antibody sequences using AbMPNN, the model weights were first obtained by downloading them from the official AbMPNN repository (https://zenodo.org/records/8164693). After downloading the weights, they were copied to the appropriate directory, specifically the “vanilla_model_weights” folder within the ProteinMPNN project.

Once the model weights were properly placed, the design process proceeded in a manner similar to ProteinMPNN. The modified “submit_example_4_non_fixed.sh” script was utilized, which was originally used for ProteinMPNN, to run the design process. This script was adjusted to specify the use of the AbMPNN model weights by updating the relevant configuration parameters.

In addition to selecting the AbMPNN model, the following parameters were maintained as in the ProteinMPNN setup:

- Number of Sequences per Target (–num_seq_per_target): Set to 32 768
- Backbone Noise (–backbone_noise): Set to 0.5
- Sampling Temperature (–sampling_temp): Maintained at 0.1

After modifying the script to specify AbMPNN's model weights and adjusting the parameters, the script was executed within the required conda environment. AbMPNN generated new antibody sequences based on its trained model, which were saved for further evaluation. These newly designed sequences were then available for subsequent structural validation and optimization steps.

### Antibody Sequence Design by AntiFold

AntiFold was cloned and installed from its official GitHub repository (https://github.com/oxpig/AntiFold). The same antibody structures were provided to AntiFold, using the default parameters recommended in its GitHub documentation (num_seq_per_target = 32 768, sampling_temp = 0.2). Despite large‐scale sampling (generating 32 768 sequences) for sequence generation, most sequences were highly similar at the target positions, resulting in a limited diversity of unique antibodies, as illustrated in Figure 7.

### Selection of Antibody Sequences by Alphafold3 for Experimental Validations

Using models such as ProteinMPNN, AbMPNN, and AntiFold, the CDR1 and CDR3 regions of the huJ3 antibody and the CDR2 region of the D6 antibody were redesigned. For each model and target region, large‐scale sequence generation (32 768 sequences) was performed, ranked them using each model's internal scoring function, and selected the top 16 candidates. After removing duplicates, a set of unique sequences was obtained for each region and model (the number of unique sequences varies and is reported in Table S2, Supporting Information). These sequences were then modeled using AlphaFold3 (AF3) multimer prediction. Based on the AF3 ranking_score, the top 2 sequences from each model and region were selected for synthesis and experimental validation, as summarized in Table S2 (Supporting Information).

### Expression and Purification of Antigen Protein and Designed Nano Antibodies

Two antigen proteins gp120 and CD16A were purified by the lab as previously described.^[^ ^33^, ^38^ ^]^ Briefly, pSecTag B containing HIV consensus gp120 sequence was transfected into expi293 cells by PEI, followed by purification using Ni‐NTA chromatography. Protein purity was identified by SDS‐PAGE. For CD16A, the CD16A cDNA was fused with monomeric Fc (mFc) and subcloned into the pSectag B vector. The CD16A‐mFc was expressed similarly as gp120 and purified by Protein A resin.

Once the newly designed antibody protein sequences were confirmed, the corresponding DNA sequences were reverse‐translated and synthesized by IDT. Then all antibody genes were cloned into pcomb3X plasmid. The constructed plasmids were transformed into competent Top10 *E. coli* bacteria and then spread onto the 2YT agar plate with ampicillin selection. After overnight incubation in 37 °C, single colonies were picked and cultured, and plasmids were extracted and sequenced by Sanger sequencing (Genewiz). The correct plasmid was transformed into competent Top10 *E. coli* and cultured at 37 °C with shaking for 8 hours. IPTG (1 mm) was then added for induction and expression at 30 °C for 16 h. Protein was purified by Ni‐NTA Agarose columns (QIAGEN). The purity of all designed nanobodies was identified by SDS‐PAGE.

### Binding Property Identification

Enzyme‐linked immunosorbent assay (ELISA) was used to evaluate the binding properties of the designed antibodies. Briefly, a 96‐well plate (Costar) was coated with 200 ng well^−1^ of gp120 or 150 ng well^−1^ of CD16A antigen protein in phosphate‐buffered saline (PBS) and incubated overnight at 4 °C, then blocked with 5% milk PBS for 1 h at 37 °C. After washing four times with a 0.05% Tween‐20 PBS solution, fivefold serially diluted antibodies with a starting of 1000 nm were incubated on the plate for 1 h at 37 °C. To ascertain the binding of VH candidate antibodies, anti‐FLAG M2 horseradish peroxidase (HRP) conjugate antibody (obtained from Sigma‐Aldrich) was employed and incubated for another 1 h. Detection of binding activity was facilitated through the use of 3,3′,5,5′‐tetramethylbenzidine (TMB) substrate from Sigma‐Aldrich, and the reaction was terminated with TMB stop buffer supplied by ScyTek Laboratories. Finally, absorbance measurements were taken at a wavelength of 450 nm to quantify the binding.

### Statistical Analysis

ELISA assays were conducted in at least technical duplicate, with the mean values displayed. Error bars indicated the standard deviation. The half‐maximal binding, 50% effective ELISA concentration (EC_50_), was defined as the concentration at which the optical density (OD) reached 50% of its maximum value. The EC_50_ was obtained by fitting the OD450 nm‐antibody concentration curves with a non‐linear regression model (variable slope, four parameters) using GraphPad Prism 9.5.0.

## Conflict of Interest

The authors declare no conflict of interest.

## Author Contributions

Z.F., W.L., T.H., Y.X., and Y.C. designed the project. Z.F. and Z.‐Y.S. tested the code. Z.‐Y.S. conducted the experiments. Z.‐Y.S., Z.F., and W.L. prepared the figures and wrote the manuscript. All authors read and approved the final manuscript.

## Supporting information



Supporting Information

## Data Availability

The data that support the findings of this study are available from the corresponding author upon reasonable request.
