# Supplementary material for: AntiBMPNN: Structure‐Guided Graph Neural Networks for Precision Antibody Engineering
Source: Adv Sci (Weinh). 2025 Jun 27;12(35):e04278. doi: 10.1002/advs.202504278 (PMC12463024; doi:10.1002/advs.202504278)
Supplement: Supplementary file 1 — Supporting Information [file ADVS-12-e04278-s001.docx]

**Supporting Information**

**AntiBMPNN: Structure-Guided Graph Neural Networks for Precision Antibody Engineering**

**Ze-Yu Sun^1,2^, Jiayi Yuan^2^, Divya Jaiswal^2^, Jingxuan Ge^3^, Tianjian Liang^2^, Jiahui Wei^1^, Jinghong Cao^2^, Yulong Li^4^, Xiaojie Chu^5^, Yan Chen^1^*, Ying Xue^2,6^*, Wei Li^5^*, Tingjun Hou^3^*, and Zhiwei Feng^2,6^***

^1^College of Pharmacology Sciences, Zhejiang University of Technology, Hangzhou, P.R. China, 310014

^2^Department of Pharmaceutical Sciences, School of Pharmacy, University of Pittsburgh, Pittsburgh, Pennsylvania 15261, United States

^3^College of Pharmaceutical Sciences, Zhejiang University, Hangzhou 310058, Zhejiang, China

^4^College of Chemistry and Environmental Engineering, Sichuan University of Science and Engineering, 643000, Zigong, P. R. China.

^5^Department of Medicine, Center for AIDS Research, Division of Infectious Diseases, School of Medicine, University of Pittsburgh, Pittsburgh, Pennsylvania, 15261, United States.

^6^AI Innovation Center, School of Pharmacy, Shenzhen University of Advanced Technology, Shenzhen, 518055, P.R. China

*To whom correspondence should be addressed; Email: [chenyan2008@zjut.edu.cn](mailto:chenyan2008@zjut.edu.cn). Tel: 412-383-0423; Email: [yix49@pitt.edu](mailto:yix49@pitt.edu). Tel:412-383-3709; Email: [liwei171@pitt.edu](mailto:liwei171@pitt.edu). Tel: 412-383-4703; Email: [tingjunhou@zju.edu.cn](mailto:tingjunhou@zju.edu.cn); Tel: 0571-88208412; Email: [fengzhiwei@suat-sz.edu.cn](mailto:fengzhiwei@suat-sz.edu.cn) Tel: 86-18127854918.

**
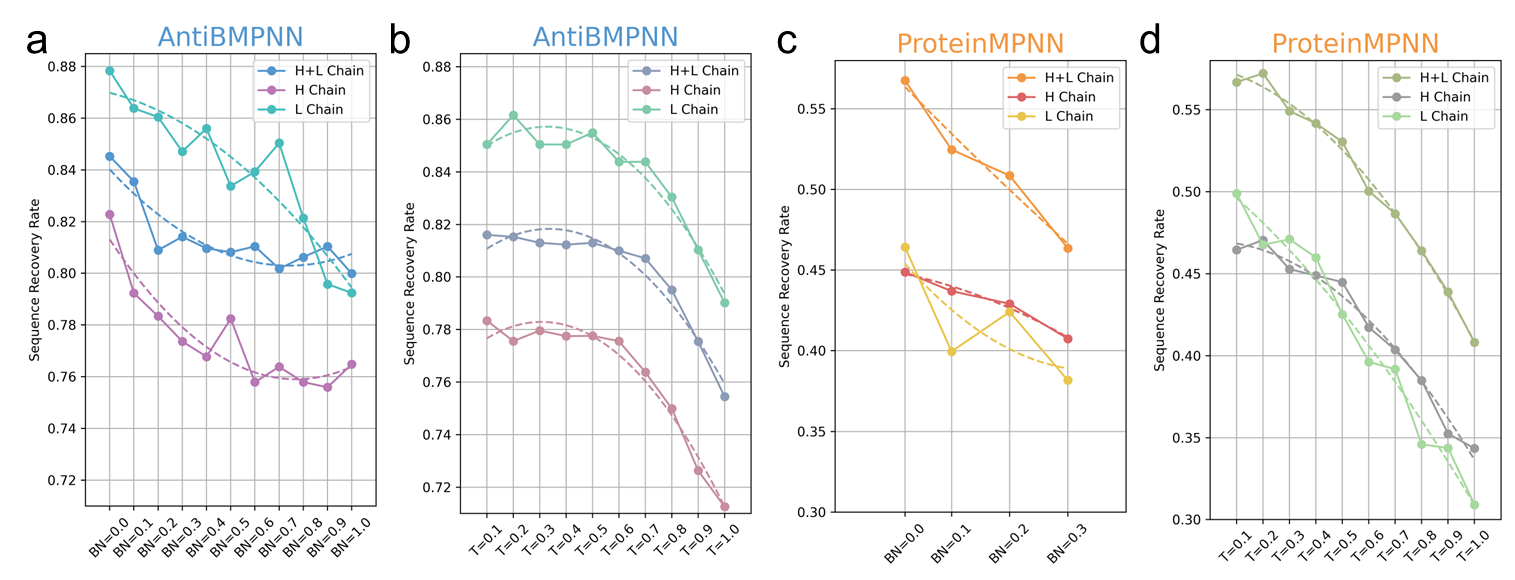
**

**Figure S1. The impact of training noise level and temperature on sequence recovery for AntiBMPNN and ProteinMPNN on the testing dataset**. To briefly illustrate the trend, We selected a representative structure to make a graph showing that as the noise of the training backbone increases or the temperature increases during inference, the average recovery rate of the sequence decreases, which means that the diversity is increasing. All structures in the test set follow this trend but the values ​​are slightly different. (a-b) The in-silico evaluation of training noise level and temperature for AntiBMPNN. (c-d) The in-silico evaluation of training noise level and temperature for ProteinMPNN. For both AntiBMPNN and ProteinMPNN, dashed lines indicate sequence recovery trends (y-axis) concerning backbone noise (BN on the x-axis) or temperature (T on the x-axis).


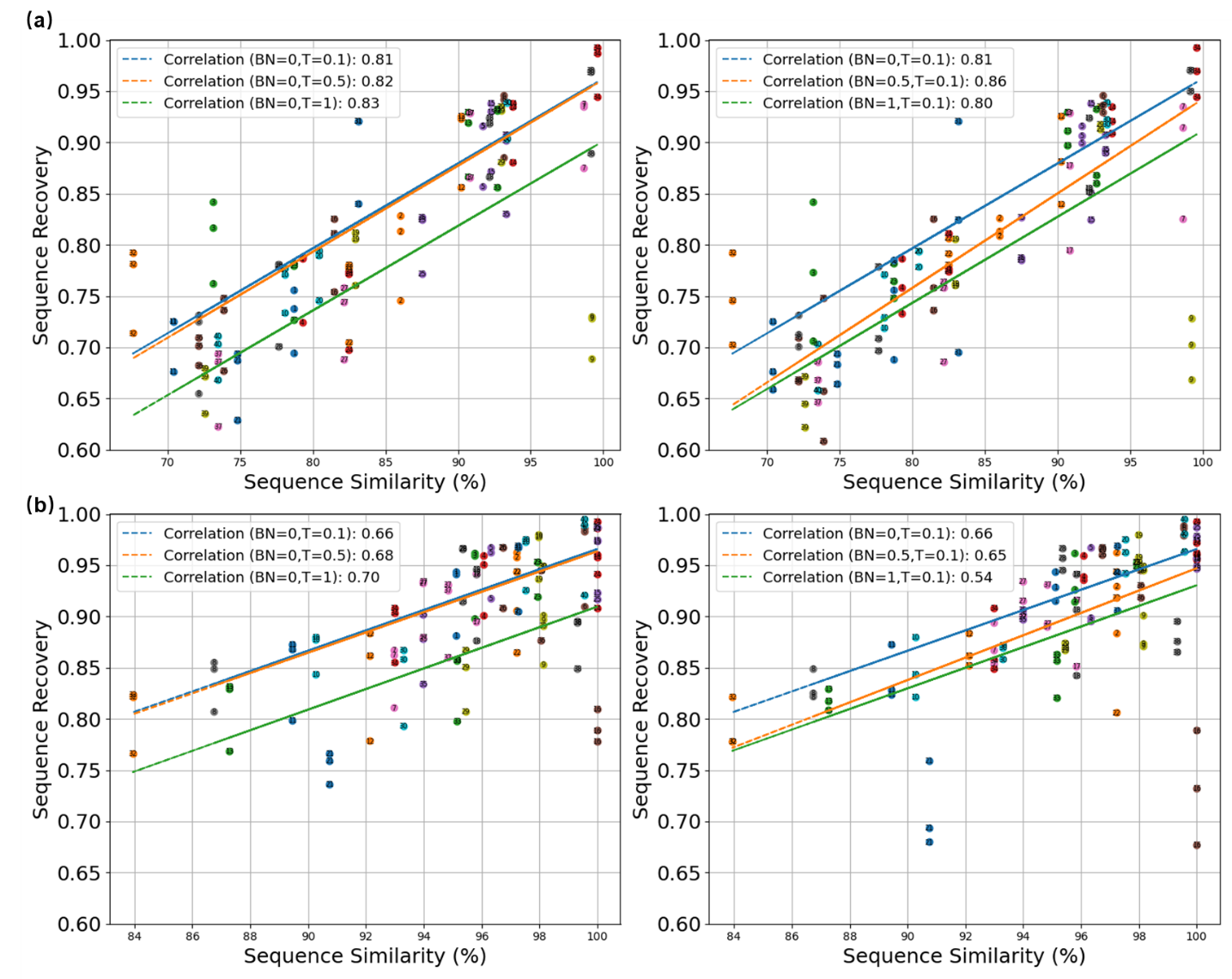


**Figure S2. The impact of sequence similarity on sequence recovery for AntiBMPNN and ProteinMPNN**. (a) The correlation between sequence recovery and similarity (from 65% to 99%) for 40 H-Chain crystal/cryo-EM structures. (b) The correlation between sequence recovery and similarity (from 84% to 100%) for 40 L-Chain crystal/cryo-EM structures. Sequence recovery is plotted on the y-axis while sequence similarity is plotted on the x-axis. Each dot labeled with a number represents a data point from an individual antibody. In the left figures of both (a) and (b), the BN (backbone noise level during training) was set to 0, while the T (temperature) was set to 0.1, 0.5, and 1, respectively. In the right figures of both (a) and (b), the BN (backbone noise level during training) was set to 0, 0.5, and 1, respectively, while the T (temperature) was set to 0.1. The number labeled inside each dot represented the structure ID.


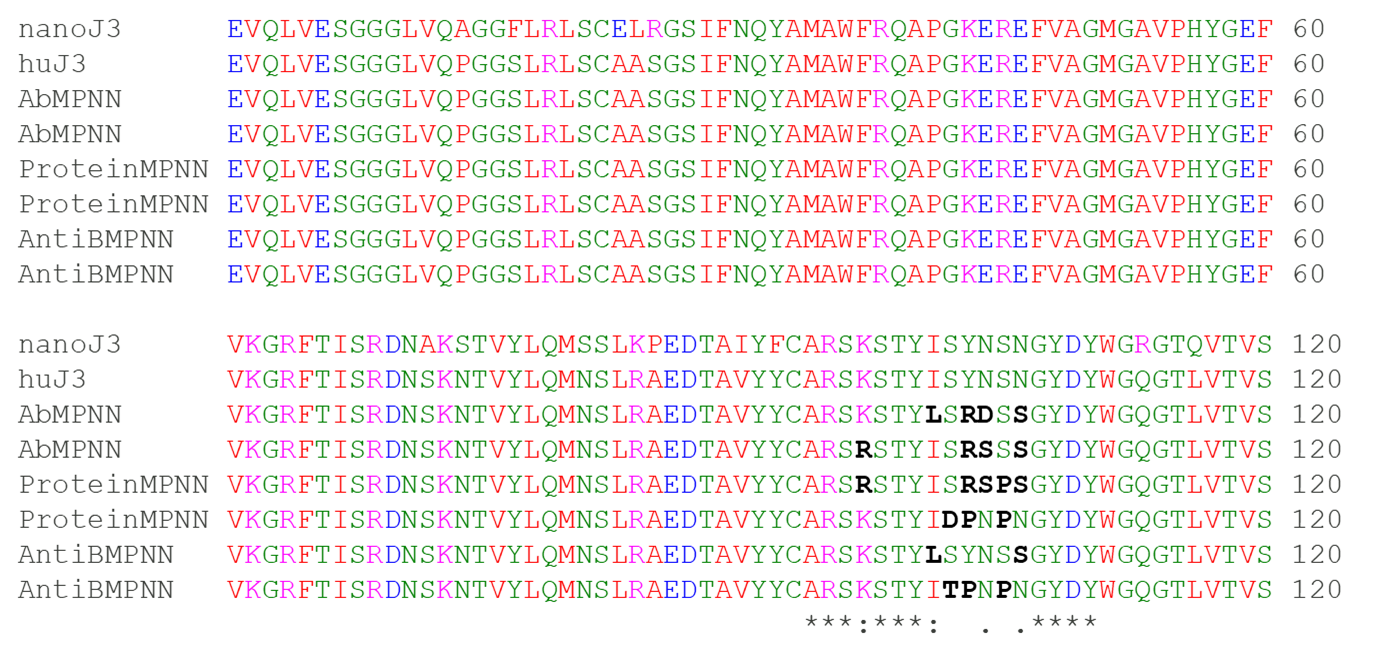


**Figure S3. Redesign of huJ3 CDR3 region (residues 94–105) using generative models.** Sequence variants of the huJ3 antibody CDR3 region (residues 94–105), generated by AntiBMPNN, ProteinMPNN, and AbMPNN while preserving the critical “STY” motif (residues 98–100). Each model proposed sequences with 2 to 5 mutations relative to the wild-type. All designed variants were experimentally tested for binding to gp120, and none exhibited measurable binding activity. This result highlights the functional sensitivity of the CDR3 region and underscores the importance of preserving key residues when comparing model performance.

**Table S1. Summary of AlphaFold3 Monomer and Multimer Modeling Results.** The table lists designed variants along with their predicted Local Distance Difference Test (pLDDT) scores from monomer modeling and ranking scores from multimer modeling. Higher scores indicate better structural confidence and predicted stability. Specifically, 3-V10 exhibited an unexpectedly low-ranking score. We determined that both a significant decrease in ipTM and a noticeable reduction in pTM contributed to the 0.26 ranking score. This suggests that the 3-V10 design is considerably less promising from the AF3 perspective. For the D6 series antibodies, since the 3D structure complex of the D6 antibody and its antigen is not available in the PDB database, it is reasonable that the ranking score is generally much lower.

| Designs | pLDDT of AlphaFold3 Monomer Modeling | Ranking Score of AlphaFold3 Multimer Modeling |
| --- | --- | --- |
| nanoJ3 | 92.77 | 0.9312 |
| HUJ3 | 91.75 | 0.9187 |
| S105P | 91.45 | 0.9179 |
| N106S | 92.03 | 0.9140 |
| N104A | 91.49 | 0.9234 |
| N104M | 91.25 | 0.9212 |
| S102N | 91.43 | 0.9230 |
| N104S | 91.65 | 0.9181 |
| Y103H | 91.34 | 0.9161 |
| Y103R | 91.50 | 0.9029 |
| 1-V1 | 92.41 | 0.9216 |
| 1-V2 | 91.95 | 0.9199 |
| 1-V3 | 91.70 | 0.9195 |
| 1-V4 | 91.52 | 0.9173 |
| 1-V5 | 92.69 | 0.9165 |
| 3-V1 | 90.87 | 0.9257 |
| 3-V2 | 91.03 | 0.9087 |
| 3-V3 | 90.82 | 0.9241 |
| 3-V4 | 91.95 | 0.9135 |
| 3-V5 | 92.01 | 0.9090 |
| 3-V6 | 91.23 | 0.9195 |
| 3-V7 | 91.71 | 0.9180 |
| 3-V8 | 92.19 | 0.9146 |
| 3-V9 | 91.51 | 0.9037 |
| 3-V10 | 91.44 | 0.2608 |
| 3-V11 | 91.29 | 0.8545 |
| D6 | 95.22 | 0.2338 |
| 2-V1 | 95.45 | 0.2307 |
| 2-V2 | 94.41 | 0.2064 |
| 2-V3 | 94.86 | 0.2052 |
| 2-V4 | 94.30 | 0.2226 |

**Table S2. Summary of design result of established protocols.** We applied AbMPNN, AntiFold, and ProteinMPNN models to design sequences targeting different regions. For each protocol, 32,768 sequences were generated and ranked using each model’s internal scoring function. The top 16 sequences were selected and filtered to obtain unique sequences (the number of unique sequences per region varies and is shown in the table). These unique sequences were then modeled using AlphaFold3 (AF3) multimer prediction with their antigen. Based on the AF3 ranking_score, the top 2 sequences from each model and region were selected for experimental validation. This table lists the sequence IDs, designed regions, design models, and the corresponding ranking_scores from AF3 multimer modeling. Sequences selected for synthesis are highlighted in bold black, representing the top two AF3-ranked sequences from each model and region.

| **Sequence ID** | **Antibody** | **Designed Region** | **Model** | **AF3 Model** | **ranking_score** | **Sequence** |
| --- | --- | --- | --- | --- | --- | --- |
| **#1** | **huJ3** | **CDR1** | **AbMPNN** | **3** | **0.9245** | **EVQLVESGGGLVQPGGSLRLSCAASGSPFSKYAMAWFRQAPGKEREFVAGMGAVPHYGEFVKGRFTISRDNSKNTVYLQMNSLRAEDTAVYYCARSKSTYISYNSNGYDYWGQGTLVTVSS** |
| **#2** | **huJ3** | **CDR1** | **AbMPNN** | **3** | **0.9206** | **EVQLVESGGGLVQPGGSLRLSCAASGSGFSSYAMAWFRQAPGKEREFVAGMGAVPHYGEFVKGRFTISRDNSKNTVYLQMNSLRAEDTAVYYCARSKSTYISYNSNGYDYWGQGTLVTVSS** |
| #3 | huJ3 | CDR1 | AbMPNN | 0 | 0.9231 | EVQLVESGGGLVQPGGSLRLSCAASGSSFSGYAMAWFRQAPGKEREFVAGMGAVPHYGEFVKGRFTISRDNSKNTVYLQMNSLRAEDTAVYYCARSKSTYISYNSNGYDYWGQGTLVTVSS |
| #4 | huJ3 | CDR1 | AbMPNN | 4 | 0.9197 | EVQLVESGGGLVQPGGSLRLSCAASGSSFSSYAMAWFRQAPGKEREFVAGMGAVPHYGEFVKGRFTISRDNSKNTVYLQMNSLRAEDTAVYYCARSKSTYISYNSNGYDYWGQGTLVTVSS |
| #5 | huJ3 | CDR1 | AbMPNN | 4 | 0.9206 | EVQLVESGGGLVQPGGSLRLSCAASGSTFSGYAMAWFRQAPGKEREFVAGMGAVPHYGEFVKGRFTISRDNSKNTVYLQMNSLRAEDTAVYYCARSKSTYISYNSNGYDYWGQGTLVTVSS |
| **#1** | **huJ3** | **CDR1** | **AntiFold** | **3** | **0.9209** | **EVQLVESGGGLVQPGGSLRLSCAASGSTFSNYAMAWFRQAPGKEREFVAGMGAVPHYGEFVKGRFTISRDNSKNTVYLQMNSLRAEDTAVYYCARSKSTYISYNSNGYDYWGQGTLVTVSS** |
| #2 | huJ3 | CDR1 | AntiFold | 0 | 0.9184 | EVQLVESGGGLVQPGGSLRLSCAASGSTFSAYAMAWFRQAPGKEREFVAGMGAVPHYGEFVKGRFTISRDNSKNTVYLQMNSLRAEDTAVYYCARSKSTYISYNSNGYDYWGQGTLVTVSS |
| #3 | huJ3 | CDR1 | AntiFold | 2 | 0.9173 | EVQLVESGGGLVQPGGSLRLSCAASGSTFTDYAMAWFRQAPGKEREFVAGMGAVPHYGEFVKGRFTISRDNSKNTVYLQMNSLRAEDTAVYYCARSKSTYISYNSNGYDYWGQGTLVTVSS |
| #4 | huJ3 | CDR1 | AntiFold | 3 | 0.9204 | EVQLVESGGGLVQPGGSLRLSCAASGSSFIHYAMAWFRQAPGKEREFVAGMGAVPHYGEFVKGRFTISRDNSKNTVYLQMNSLRAEDTAVYYCARSKSTYISYNSNGYDYWGQGTLVTVSS |
| **#5** | **huJ3** | **CDR1** | **AntiFold** | **4** | **0.9237** | **EVQLVESGGGLVQPGGSLRLSCAASGSTFDNYAMAWFRQAPGKEREFVAGMGAVPHYGEFVKGRFTISRDNSKNTVYLQMNSLRAEDTAVYYCARSKSTYISYNSNGYDYWGQGTLVTVSS** |
| #6 | huJ3 | CDR1 | AntiFold | 3 | 0.9141 | EVQLVESGGGLVQPGGSLRLSCAASGSTFHHYAMAWFRQAPGKEREFVAGMGAVPHYGEFVKGRFTISRDNSKNTVYLQMNSLRAEDTAVYYCARSKSTYISYNSNGYDYWGQGTLVTVSS |
| #7 | huJ3 | CDR1 | AntiFold | 4 | 0.9147 | EVQLVESGGGLVQPGGSLRLSCAASGSTFDTYAMAWFRQAPGKEREFVAGMGAVPHYGEFVKGRFTISRDNSKNTVYLQMNSLRAEDTAVYYCARSKSTYISYNSNGYDYWGQGTLVTVSS |
| #8 | huJ3 | CDR1 | AntiFold | 4 | 0.9124 | EVQLVESGGGLVQPGGSLRLSCAASGSIFDSYAMAWFRQAPGKEREFVAGMGAVPHYGEFVKGRFTISRDNSKNTVYLQMNSLRAEDTAVYYCARSKSTYISYNSNGYDYWGQGTLVTVSS |
| #9 | huJ3 | CDR1 | AntiFold | 3 | 0.9195 | EVQLVESGGGLVQPGGSLRLSCAASGSSFNSYAMAWFRQAPGKEREFVAGMGAVPHYGEFVKGRFTISRDNSKNTVYLQMNSLRAEDTAVYYCARSKSTYISYNSNGYDYWGQGTLVTVSS |
| #10 | huJ3 | CDR1 | AntiFold | 4 | 0.9171 | EVQLVESGGGLVQPGGSLRLSCAASGSIFNDYAMAWFRQAPGKEREFVAGMGAVPHYGEFVKGRFTISRDNSKNTVYLQMNSLRAEDTAVYYCARSKSTYISYNSNGYDYWGQGTLVTVSS |
| #1 | huJ3 | CDR1 | ProteinMPNN | 0 | 0.9205 | EVQLVESGGGLVQPGGSLRLSCAASGSPFAGYAMAWFRQAPGKEREFVAGMGAVPHYGEFVKGRFTISRDNSKNTVYLQMNSLRAEDTAVYYCARSKSTYISYNSNGYDYWGQGTLVTVSS |
| #2 | huJ3 | CDR1 | ProteinMPNN | 3 | 0.9159 | EVQLVESGGGLVQPGGSLRLSCAASGSSFGTYAMAWFRQAPGKEREFVAGMGAVPHYGEFVKGRFTISRDNSKNTVYLQMNSLRAEDTAVYYCARSKSTYISYNSNGYDYWGQGTLVTVSS |
| #3 | huJ3 | CDR1 | ProteinMPNN | 1 | 0.9178 | EVQLVESGGGLVQPGGSLRLSCAASGSAFGGYAMAWFRQAPGKEREFVAGMGAVPHYGEFVKGRFTISRDNSKNTVYLQMNSLRAEDTAVYYCARSKSTYISYNSNGYDYWGQGTLVTVSS |
| #4 | huJ3 | CDR1 | ProteinMPNN | 0 | 0.9218 | EVQLVESGGGLVQPGGSLRLSCAASGSDFGGYAMAWFRQAPGKEREFVAGMGAVPHYGEFVKGRFTISRDNSKNTVYLQMNSLRAEDTAVYYCARSKSTYISYNSNGYDYWGQGTLVTVSS |
| **#5** | **huJ3** | **CDR1** | **ProteinMPNN** | **4** | **0.9222** | **EVQLVESGGGLVQPGGSLRLSCAASGSGFGSYAMAWFRQAPGKEREFVAGMGAVPHYGEFVKGRFTISRDNSKNTVYLQMNSLRAEDTAVYYCARSKSTYISYNSNGYDYWGQGTLVTVSS** |
| #6 | huJ3 | CDR1 | ProteinMPNN | 0 | 0.9155 | EVQLVESGGGLVQPGGSLRLSCAASGSSFPGYAMAWFRQAPGKEREFVAGMGAVPHYGEFVKGRFTISRDNSKNTVYLQMNSLRAEDTAVYYCARSKSTYISYNSNGYDYWGQGTLVTVSS |
| #7 | huJ3 | CDR1 | ProteinMPNN | 0 | 0.9203 | EVQLVESGGGLVQPGGSLRLSCAASGSSFGGYAMAWFRQAPGKEREFVAGMGAVPHYGEFVKGRFTISRDNSKNTVYLQMNSLRAEDTAVYYCARSKSTYISYNSNGYDYWGQGTLVTVSS |
| **#8** | **huJ3** | **CDR1** | **ProteinMPNN** | **4** | **0.9230** | **EVQLVESGGGLVQPGGSLRLSCAASGSDFKTYAMAWFRQAPGKEREFVAGMGAVPHYGEFVKGRFTISRDNSKNTVYLQMNSLRAEDTAVYYCARSKSTYISYNSNGYDYWGQGTLVTVSS** |
| #9 | huJ3 | CDR1 | ProteinMPNN | 3 | 0.9219 | EVQLVESGGGLVQPGGSLRLSCAASGSGFTGYAMAWFRQAPGKEREFVAGMGAVPHYGEFVKGRFTISRDNSKNTVYLQMNSLRAEDTAVYYCARSKSTYISYNSNGYDYWGQGTLVTVSS |
| #10 | huJ3 | CDR1 | ProteinMPNN | 4 | 0.9210 | EVQLVESGGGLVQPGGSLRLSCAASGSEFGGYAMAWFRQAPGKEREFVAGMGAVPHYGEFVKGRFTISRDNSKNTVYLQMNSLRAEDTAVYYCARSKSTYISYNSNGYDYWGQGTLVTVSS |
| #11 | huJ3 | CDR1 | ProteinMPNN | 0 | 0.9210 | EVQLVESGGGLVQPGGSLRLSCAASGSPFKGYAMAWFRQAPGKEREFVAGMGAVPHYGEFVKGRFTISRDNSKNTVYLQMNSLRAEDTAVYYCARSKSTYISYNSNGYDYWGQGTLVTVSS |
| #12 | huJ3 | CDR1 | ProteinMPNN | 0 | 0.9148 | EVQLVESGGGLVQPGGSLRLSCAASGSPFGGYAMAWFRQAPGKEREFVAGMGAVPHYGEFVKGRFTISRDNSKNTVYLQMNSLRAEDTAVYYCARSKSTYISYNSNGYDYWGQGTLVTVSS |
| **#1** | **D6** | **CDR2** | **AbMPNN** | **2** | **0.2336** | **EVQLVESGGGLVQPGGSLRLSCAASGFTFSNYGMSWVRQAPGKGLEWIGTIYYSGSTNYNPSLKSGVTISRDNSKNTLYLQMNSLRAEDTATYYCARESIDYWGQGTLVTVSS** |
| **#2** | **D6** | **CDR2** | **AbMPNN** | **4** | **0.2351** | **EVQLVESGGGLVQPGGSLRLSCAASGFTFSNYGMSWVRQAPGKGLEWIGLIYYSGSTNYNPSLKSGVTISRDNSKNTLYLQMNSLRAEDTATYYCARESIDYWGQGTLVTVSS** |
| #3 | D6 | CDR2 | AbMPNN | 4 | 0.2236 | EVQLVESGGGLVQPGGSLRLSCAASGFTFSNYGMSWVRQAPGKGLEWIGTIYYSGSTNYNPSLKSRVTISRDNSKNTLYLQMNSLRAEDTATYYCARESIDYWGQGTLVTVSS |
| #4 | D6 | CDR2 | AbMPNN | 2 | 0.2070 | EVQLVESGGGLVQPGGSLRLSCAASGFTFSNYGMSWVRQAPGKGLEWIGYIYYSGSTNYNPSLKSNVTISRDNSKNTLYLQMNSLRAEDTATYYCARESIDYWGQGTLVTVSS |
| #5 | D6 | CDR2 | AbMPNN | 4 | 0.2066 | EVQLVESGGGLVQPGGSLRLSCAASGFTFSNYGMSWVRQAPGKGLEWIGYIYYSGSTNYNPSLKSRVTISRDNSKNTLYLQMNSLRAEDTATYYCARESIDYWGQGTLVTVSS |
| #6 | D6 | CDR2 | AbMPNN | 4 | 0.2103 | EVQLVESGGGLVQPGGSLRLSCAASGFTFSNYGMSWVRQAPGKGLEWIGYIYYSGSTNYNPSLKSSVTISRDNSKNTLYLQMNSLRAEDTATYYCARESIDYWGQGTLVTVSS |
| #1 | D6 | CDR2 | AntiFold | 0 | 0.2292 | EVQLVESGGGLVQPGGSLRLSCAASGFTFSNYGMSWVRQAPGKGLEWIGDIYYSGSTNYNPSLKSRVTISRDNSKNTLYLQMNSLRAEDTATYYCARESIDYWGQGTLVTVSS |
| #2 | D6 | CDR2 | AntiFold | 0 | 0.2313 | EVQLVESGGGLVQPGGSLRLSCAASGFTFSNYGMSWVRQAPGKGLEWIGEIYYSGSTNYNPSLKSRVTISRDNSKNTLYLQMNSLRAEDTATYYCARESIDYWGQGTLVTVSS |
| #3 | D6 | CDR2 | AntiFold | 2 | 0.2545 | EVQLVESGGGLVQPGGSLRLSCAASGFTFSNYGMSWVRQAPGKGLEWIGGIYYSGSTNYNPSLKSRVTISRDNSKNTLYLQMNSLRAEDTATYYCARESIDYWGQGTLVTVSS |
| #4 | D6 | CDR2 | AntiFold | 0 | 0.2252 | EVQLVESGGGLVQPGGSLRLSCAASGFTFSNYGMSWVRQAPGKGLEWIGHIYYSGSTNYNPSLKSRVTISRDNSKNTLYLQMNSLRAEDTATYYCARESIDYWGQGTLVTVSS |
| **#5** | **D6** | **CDR2** | **AntiFold** | **0** | **0.3047** | **EVQLVESGGGLVQPGGSLRLSCAASGFTFSNYGMSWVRQAPGKGLEWIGLIYYSGSTNYNPSLKSRVTISRDNSKNTLYLQMNSLRAEDTATYYCARESIDYWGQGTLVTVSS** |
| #6 | D6 | CDR2 | AntiFold | 4 | 0.2135 | EVQLVESGGGLVQPGGSLRLSCAASGFTFSNYGMSWVRQAPGKGLEWIGMIYYSGSTNYNPSLKSRVTISRDNSKNTLYLQMNSLRAEDTATYYCARESIDYWGQGTLVTVSS |
| **#7** | **D6** | **CDR2** | **AntiFold** | **4** | **0.2613** | **EVQLVESGGGLVQPGGSLRLSCAASGFTFSNYGMSWVRQAPGKGLEWIGSIYYSGSTNYNPSLKSRVTISRDNSKNTLYLQMNSLRAEDTATYYCARESIDYWGQGTLVTVSS** |
| #1 | D6 | CDR2 | ProteinMPNN | 2 | 0.2540 | EVQLVESGGGLVQPGGSLRLSCAASGFTFSNYGMSWVRQAPGKGLEWIGGIYYSGSTNYNPSLKSGVTISRDNSKNTLYLQMNSLRAEDTATYYCARESIDYWGQGTLVTVSS |
| #2 | D6 | CDR2 | ProteinMPNN | 3 | 0.2787 | EVQLVESGGGLVQPGGSLRLSCAASGFTFSNYGMSWVRQAPGKGLEWIGGIYYSGSTNYNPSLKSSVTISRDNSKNTLYLQMNSLRAEDTATYYCARESIDYWGQGTLVTVSS |
| #3 | D6 | CDR2 | ProteinMPNN | 4 | 0.2341 | EVQLVESGGGLVQPGGSLRLSCAASGFTFSNYGMSWVRQAPGKGLEWIGTIYYSGSTNYNPSLKSTVTISRDNSKNTLYLQMNSLRAEDTATYYCARESIDYWGQGTLVTVSS |
| **#4** | **D6** | **CDR2** | **ProteinMPNN** | **0** | **0.2984** | **EVQLVESGGGLVQPGGSLRLSCAASGFTFSNYGMSWVRQAPGKGLEWIGSIYYSGSTNYNPSLKSGVTISRDNSKNTLYLQMNSLRAEDTATYYCARESIDYWGQGTLVTVSS** |
| #5 | D6 | CDR2 | ProteinMPNN | 3 | 0.2503 | EVQLVESGGGLVQPGGSLRLSCAASGFTFSNYGMSWVRQAPGKGLEWIGSIYYSGSTNYNPSLKSLVTISRDNSKNTLYLQMNSLRAEDTATYYCARESIDYWGQGTLVTVSS |
| #6 | D6 | CDR2 | ProteinMPNN | 4 | 0.2422 | EVQLVESGGGLVQPGGSLRLSCAASGFTFSNYGMSWVRQAPGKGLEWIGSIYYSGSTNYNPSLKSAVTISRDNSKNTLYLQMNSLRAEDTATYYCARESIDYWGQGTLVTVSS |
| **#7** | **D6** | **CDR2** | **ProteinMPNN** | **3** | **0.2790** | **EVQLVESGGGLVQPGGSLRLSCAASGFTFSNYGMSWVRQAPGKGLEWIGIIYYSGSTNYNPSLKSKVTISRDNSKNTLYLQMNSLRAEDTATYYCARESIDYWGQGTLVTVSS** |
| #1 | huJ3 | CDR3 | AbMPNN | 1 | 0.9096 | EVQLVESGGGLVQPGGSLRLSCAASGSIFNQYAMAWFRQAPGKEREFVAGMGAVPHYGEFVKGRFTISRDNSKNTVYLQMNSLRAEDTAVYYCARSSSTYIGYNSSGYDYWGQGTLVTVSS |
| **#2** | **huJ3** | **CDR3** | **AbMPNN** | **1** | **0.9136** | **EVQLVESGGGLVQPGGSLRLSCAASGSIFNQYAMAWFRQAPGKEREFVAGMGAVPHYGEFVKGRFTISRDNSKNTVYLQMNSLRAEDTAVYYCARSSSTYISYNSSGYDYWGQGTLVTVSS** |
| #3 | huJ3 | CDR3 | AbMPNN | 4 | 0.9087 | EVQLVESGGGLVQPGGSLRLSCAASGSIFNQYAMAWFRQAPGKEREFVAGMGAVPHYGEFVKGRFTISRDNSKNTVYLQMNSLRAEDTAVYYCARSSSTYIYYNSSGYDYWGQGTLVTVSS |
| #4 | huJ3 | CDR3 | AbMPNN | 0 | 0.9106 | EVQLVESGGGLVQPGGSLRLSCAASGSIFNQYAMAWFRQAPGKEREFVAGMGAVPHYGEFVKGRFTISRDNSKNTVYLQMNSLRAEDTAVYYCARSSSTYISYNSGGYDYWGQGTLVTVSS |
| #5 | huJ3 | CDR3 | AbMPNN | 4 | 0.9096 | EVQLVESGGGLVQPGGSLRLSCAASGSIFNQYAMAWFRQAPGKEREFVAGMGAVPHYGEFVKGRFTISRDNSKNTVYLQMNSLRAEDTAVYYCARSGSTYIYYNSSGYDYWGQGTLVTVSS |
| #6 | huJ3 | CDR3 | AbMPNN | 4 | 0.9106 | EVQLVESGGGLVQPGGSLRLSCAASGSIFNQYAMAWFRQAPGKEREFVAGMGAVPHYGEFVKGRFTISRDNSKNTVYLQMNSLRAEDTAVYYCARSGSTYIGYNSSGYDYWGQGTLVTVSS |
| **#7** | **huJ3** | **CDR3** | **AbMPNN** | **4** | **0.9118** | **EVQLVESGGGLVQPGGSLRLSCAASGSIFNQYAMAWFRQAPGKEREFVAGMGAVPHYGEFVKGRFTISRDNSKNTVYLQMNSLRAEDTAVYYCARSGSTYIYYNSGGYDYWGQGTLVTVSS** |
| **#1** | **huJ3** | **CDR3** | **AntiFold** | **3** | **0.8922** | **EVQLVESGGGLVQPGGSLRLSCAASGSIFNQYAMAWFRQAPGKEREFVAGMGAVPHYGEFVKGRFTISRDNSKNTVYLQMNSLRAEDTAVYYCARSRSTYISYNSDGYDYWGQGTLVTVSS** |
| #2 | huJ3 | CDR3 | AntiFold | 3 | 0.8847 | EVQLVESGGGLVQPGGSLRLSCAASGSIFNQYAMAWFRQAPGKEREFVAGMGAVPHYGEFVKGRFTISRDNSKNTVYLQMNSLRAEDTAVYYCARSSSTYITYNSDGYDYWGQGTLVTVSS |
| #3 | huJ3 | CDR3 | AntiFold | 0 | 0.8913 | EVQLVESGGGLVQPGGSLRLSCAASGSIFNQYAMAWFRQAPGKEREFVAGMGAVPHYGEFVKGRFTISRDNSKNTVYLQMNSLRAEDTAVYYCARSKSTYISYNSDGYDYWGQGTLVTVSS |
| #4 | huJ3 | CDR3 | AntiFold | 0 | 0.8903 | EVQLVESGGGLVQPGGSLRLSCAASGSIFNQYAMAWFRQAPGKEREFVAGMGAVPHYGEFVKGRFTISRDNSKNTVYLQMNSLRAEDTAVYYCARSLSTYIGYNSDGYDYWGQGTLVTVSS |
| #5 | huJ3 | CDR3 | AntiFold | 0 | 0.8904 | EVQLVESGGGLVQPGGSLRLSCAASGSIFNQYAMAWFRQAPGKEREFVAGMGAVPHYGEFVKGRFTISRDNSKNTVYLQMNSLRAEDTAVYYCARSNSTYISYNSDGYDYWGQGTLVTVSS |
| **#6** | **huJ3** | **CDR3** | **AntiFold** | **0** | **0.8981** | **EVQLVESGGGLVQPGGSLRLSCAASGSIFNQYAMAWFRQAPGKEREFVAGMGAVPHYGEFVKGRFTISRDNSKNTVYLQMNSLRAEDTAVYYCARSLSTYISYNSDGYDYWGQGTLVTVSS** |
| #7 | huJ3 | CDR3 | AntiFold | 1 | 0.8587 | EVQLVESGGGLVQPGGSLRLSCAASGSIFNQYAMAWFRQAPGKEREFVAGMGAVPHYGEFVKGRFTISRDNSKNTVYLQMNSLRAEDTAVYYCARSFSTYIDYNSDGYDYWGQGTLVTVSS |
| #1 | huJ3 | CDR3 | ProteinMPNN | 4 | 0.9025 | EVQLVESGGGLVQPGGSLRLSCAASGSIFNQYAMAWFRQAPGKEREFVAGMGAVPHYGEFVKGRFTISRDNSKNTVYLQMNSLRAEDTAVYYCARSKSTYIPYNSGGYDYWGQGTLVTVSS |
| #2 | huJ3 | CDR3 | ProteinMPNN | 4 | 0.9103 | EVQLVESGGGLVQPGGSLRLSCAASGSIFNQYAMAWFRQAPGKEREFVAGMGAVPHYGEFVKGRFTISRDNSKNTVYLQMNSLRAEDTAVYYCARSGSTYILYNSGGYDYWGQGTLVTVSS |
| #3 | huJ3 | CDR3 | ProteinMPNN | 4 | 0.9059 | EVQLVESGGGLVQPGGSLRLSCAASGSIFNQYAMAWFRQAPGKEREFVAGMGAVPHYGEFVKGRFTISRDNSKNTVYLQMNSLRAEDTAVYYCARSGSTYIAYNSGGYDYWGQGTLVTVSS |
| #4 | huJ3 | CDR3 | ProteinMPNN | 0 | 0.9090 | EVQLVESGGGLVQPGGSLRLSCAASGSIFNQYAMAWFRQAPGKEREFVAGMGAVPHYGEFVKGRFTISRDNSKNTVYLQMNSLRAEDTAVYYCARSGSTYIGYNSAGYDYWGQGTLVTVSS |
| #5 | huJ3 | CDR3 | ProteinMPNN | 4 | 0.9085 | EVQLVESGGGLVQPGGSLRLSCAASGSIFNQYAMAWFRQAPGKEREFVAGMGAVPHYGEFVKGRFTISRDNSKNTVYLQMNSLRAEDTAVYYCARSTSTYIGYNSGGYDYWGQGTLVTVSS |
| **#6** | **huJ3** | **CDR3** | **ProteinMPNN** | **3** | **0.9106** | **EVQLVESGGGLVQPGGSLRLSCAASGSIFNQYAMAWFRQAPGKEREFVAGMGAVPHYGEFVKGRFTISRDNSKNTVYLQMNSLRAEDTAVYYCARSGSTYIDYNSAGYDYWGQGTLVTVSS** |
| #7 | huJ3 | CDR3 | ProteinMPNN | 3 | 0.9025 | EVQLVESGGGLVQPGGSLRLSCAASGSIFNQYAMAWFRQAPGKEREFVAGMGAVPHYGEFVKGRFTISRDNSKNTVYLQMNSLRAEDTAVYYCARSLSTYIGYNSGGYDYWGQGTLVTVSS |
| #8 | huJ3 | CDR3 | ProteinMPNN | 4 | 0.9048 | EVQLVESGGGLVQPGGSLRLSCAASGSIFNQYAMAWFRQAPGKEREFVAGMGAVPHYGEFVKGRFTISRDNSKNTVYLQMNSLRAEDTAVYYCARSGSTYIPYNSEGYDYWGQGTLVTVSS |
| **#9** | **huJ3** | **CDR3** | **ProteinMPNN** | **0** | **0.9122** | **EVQLVESGGGLVQPGGSLRLSCAASGSIFNQYAMAWFRQAPGKEREFVAGMGAVPHYGEFVKGRFTISRDNSKNTVYLQMNSLRAEDTAVYYCARSKSTYIFYNSGGYDYWGQGTLVTVSS** |
| #10 | huJ3 | CDR3 | ProteinMPNN | 3 | 0.9029 | EVQLVESGGGLVQPGGSLRLSCAASGSIFNQYAMAWFRQAPGKEREFVAGMGAVPHYGEFVKGRFTISRDNSKNTVYLQMNSLRAEDTAVYYCARSGSTYIPYNSTGYDYWGQGTLVTVSS |
| #11 | huJ3 | CDR3 | ProteinMPNN | 3 | 0.8912 | EVQLVESGGGLVQPGGSLRLSCAASGSIFNQYAMAWFRQAPGKEREFVAGMGAVPHYGEFVKGRFTISRDNSKNTVYLQMNSLRAEDTAVYYCARSGSTYIPYNSGGYDYWGQGTLVTVSS |
| #12 | huJ3 | CDR3 | ProteinMPNN | 3 | 0.9024 | EVQLVESGGGLVQPGGSLRLSCAASGSIFNQYAMAWFRQAPGKEREFVAGMGAVPHYGEFVKGRFTISRDNSKNTVYLQMNSLRAEDTAVYYCARSGSTYIEYNSGGYDYWGQGTLVTVSS |
